# Design and Synthesis of Marine-Inspired Itampolin A Derivatives to Overcome Chemoresistance in NSCLC via Cholesterol Homeostasis Modulation

**DOI:** 10.3390/md23090357

**Published:** 2025-09-15

**Authors:** Hai-Ying Zhang, Shun-Chang Ji, Si-Hua Xie, Yu Chen, Cai-Xia Lin, Xu Huang, Yi-Qiao Wang, Jing-Wei Liang, Yan Liu

**Affiliations:** 1 Engineering Research Center of Tropical Medicine Innovation and Transformation of Ministry of Education, School of Pharmacy, Hainan Medical University, Haikou 571199, China; 2 Hainan Provincial Key Laboratory for Research and Development of Tropical Herbs, Haikou 571199, China; 3 School of Pharmacy, China Medical University, Shenyang 110122, China

**Keywords:** non-small cell lung cancer, drug-resistant, MAPK pathway, cholesterol, bioinformatic

## Abstract

Recent studies on brominated tyrosine-derived marine natural products have significantly expanded the library of known structures and revealed their potent and diverse antitumor mechanisms. Building upon our previous research on the natural product itampolin A isolated from marine sponges, we conducted structural optimizations and explored the structure–-activity relationships (SARs) of novel scaffold derivatives concerning their inhibitory activities against lung cancer cells. In the present study, we further synthesized 15 novel derivatives, and compound **4l** demonstrated selective anti-proliferative activity against gefitinib-resistant PC9/GR cells, showing 4-fold greater potency compared to parental PC9 cells. Building on this finding, the present study aims to investigate the molecular mechanisms underlying the anti-proliferative effects of 4l in drug-resistant NSCLC models. Through cell cycle analysis, apoptosis assays, and signaling pathway evaluation, we seek to establish a theoretical foundation for developing novel therapeutic agents against chemotherapy-resistant lung cancer.

## 1. Introduction

Marine organisms, owing to their unique survival environments and evolutionary strategies, serve as a treasure trove of natural bioactive compounds. As one of the oldest multicellular animal groups, sponges have garnered significant attention for their remarkable chemical diversity and biological activities [1]. Through long-term ecological adaptation, sponges have evolved complex secondary metabolic pathways to defend against predators, pathogens, and environmental stressors, synthesizing a vast array of structurally novel compounds, including alkaloids, terpenoids, peptides, and polyketides. These molecules exhibit not only antimicrobial, antiviral, anti-inflammatory, and immunomodulatory properties but also demonstrate immense potential in treating cancers, neurological disorders, and other diseases [2,3]. For instance, cytarabine, a compound isolated from sponges, has become a first-line clinical drug for leukemia, while the Halichondrin B derivative eribulin is utilized in chemotherapy for advanced breast cancer [4,5]. In recent years, advancements in deep-sea exploration and synthetic biology technologies have further expanded the scope of drug development by uncovering metabolites from sponge-associated microorganisms. However, despite their promise, the exploitation of marine sponge-derived bioactive components faces challenges such as resource sustainability, structural complexity of compounds, and incomplete understanding of their mechanisms of action [2,6].

**Figure 1:**
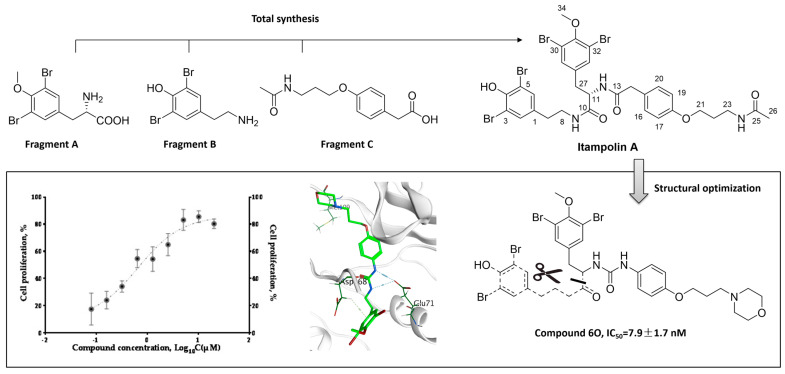
Total synthesis of itampolin A and its preliminary structural optimization.

The bromotyrosine alkaloid itampolin A (Figure 1), isolated from sponges of the genus *Iotrochota*, exhibits a broad spectrum of biological activities. Its structure features a unique trimeric assembly of brominated tyrosine units. Our research has achieved the first total synthesis of Itampolin A, enabling its scalable production independent of the limited natural supply [7]. Subsequent studies revealed significant anti-lung cancer activity for itampolin A. Structural optimization efforts led to derivatives with enhanced anti-cancer efficacy [8]. Notably, certain structurally simplified derivatives, lacking the bromotyramine fragment (Fragment B), demonstrated surprising and selective inhibitory effects against the proliferation of drug-resistant lung cancer cells. This finding highlights the potential for developing novel agents capable of overcoming chemotherapy resistance.

Lung cancer remains one of the most formidable challenges in oncology today, with chemotherapy resistance constituting a major barrier to successful treatment [9,10]. Clinical data indicate that nearly half of non-small cell lung cancer (NSCLC) patients develop intrinsic or acquired resistance to first-line chemotherapy and targeted agents, ultimately leading to disease recurrence and mortality [11,12,13,14,15]. This multifaceted resistance arises through diverse mechanisms, including gene mutations, upregulation of drug efflux pumps, activation of compensatory survival pathways, and metabolic reprogramming [16,17,18]. The complexity of these resistance mechanisms underscores the critical need for novel chemotherapeutic agents capable of selectively targeting resistant clones or disrupting adaptive resistance networks [19,20]. However, traditional drug discovery pipelines have proven inadequate in addressing the rapid evolution of resistance mechanisms [18,21]. This therapeutic gap highlights the imperative to explore unconventional chemical scaffolds, particularly those derived from marine organisms mentioned above. Such compounds offer significant potential as they often possess inherent bioactive properties and have evolved under selective pressures conducive to overcoming environmental stressors, including cytotoxic challenges.

Based on our prior discovery of itampolin A and its derivatives, they inhibited mitogen-activated protein (MAPK) kinase signaling pathway by targeting p38α in lung cancer cells. This study focuses on the rational design and synthesis of novel itampolin A derivatives, leveraging the targeted deletion of Fragment B. The aim is to develop optimized analogs with enhanced efficacy against drug-resistant NSCLC models. Comprehensive biological evaluation of these marine-derived compounds was conducted using an orthogonal pharmacological approach, encompassing phenotypic screening, apoptosis profiling, and transcriptomic analysis. The underlying molecular mechanisms enabling them to overcome drug resistance were systematically elucidated. This research not only advances the development of marine natural products but also provides critical insights for addressing the clinical challenge of refractory lung cancer therapy.

## 2. Results

### 2.1. Fragment-Based Drug Design

P38 MAPK is a member of the serine/threonine protein kinase superfamily, exhibits a three-dimensional structure and domain organization highly conserved among other MAPKs, yet possesses distinct regulatory characteristics [22]. The functional activity of p38α is an isomer of p38 MAPK, which is highly expressed in lung cancer, fundamentally a dynamic process involving protein–protein interactions [23]. Precise regulation of its kinase activity, substrate specificity, subcellular localization, and signal output relies not solely on its intrinsic catalytic domain but is predominantly governed by the formation of transient protein complexes and associated conformational changes [23,24]. Conventional inhibitors targeting only the ATP-binding pocket can block catalytic activity but fail to precisely intervene in p38α conformational selectivity and spatiotemporal specificity within distinct signaling pathways [25,26,27]. Understanding the structural domains of p38α is therefore fundamental to elucidating its function, regulatory mechanisms, and designing specific inhibitors.

**Figure 2:**
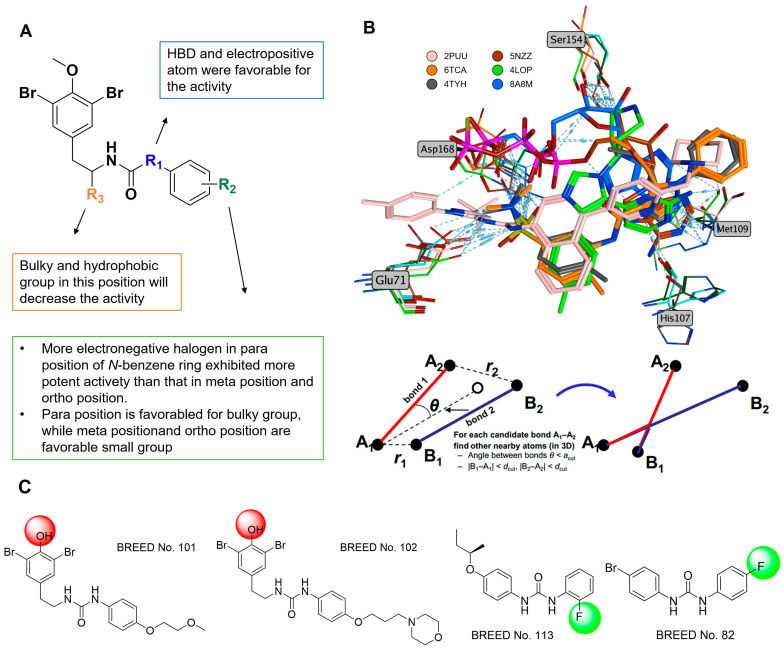
(**A**) SAR of brominated tyrosine derivatives in our previous work. (**B**) Workflow of the BREED method: p38α inhibitors were structurally aligned based on the receptor component, then ligand fragmentation based on interatomic distance and bonding angle. (**C**) The representative molecules generated by the BREED process.

In our prior study, we investigated the structure–activity relationship (SAR) of brominated tyrosine derivatives utilizing itampolin A as a lead compound. It was found that the conversion of the carbon atom at the 14-position to a nitrogen atom contributed to enhancing the activity of the derivatives. Furthermore, cleavage of the fragment B improved activity against the p38α (Figure 2A). Research indicates that during complex formation with other proteins, the G-loop of p38α undergoes inward displacement; this loop constitutes part of the kinase’s active site. Consequently, the active site pocket becomes slightly constricted, accompanied by changes in the amino acid residues lining its entrance.

Therefore, this study aims to further optimize the p38α inhibitors identified in our previous work, specifically addressing the aforementioned conformational changes. We collected and curated all available three-dimensional structures of p38α in complex with other proteins from the Protein Data Bank (PDB); these structures were structurally aligned based on the receptor component. We employed BREED technology, a Fragment-Based Drug Design (FBDD) methodology. This approach leverages a set of aligned 3D ligand structures bound to the same target or target family, operating through two principal steps: (1) alignment of ligands, followed by (2) fragmentation of ligands based on interatomic distances and bond angles (Figure 2B). A scoring scheme then assigns individual scores to each fragment. Guided by these fragment scores, a greedy search algorithm incrementally constructs novel ligands. Subsequently, these generated small molecules were screened using pharmacophore models and Lipinski’s Rule of Five (RO5) criteria. The BREED results generated by the MOE software are detailed in Supplementary Table S1. It was observed that some generated molecules retained the scaffold of compound **6o**. Interestingly, however, the methyl groups of the methoxy substituents on fragment A were consistently trimmed off during the BREED process. It indicates that the absence of methyl groups may be more in line with the molecular dynamic characteristics of p38α (Figure 2C). Furthermore, we discovered that many generated molecules contained fluorine atoms. Although these compounds did not fully comply with the Rule of Five (RO5), they nonetheless provided critical insights into our drug design efforts.

### 2.2. Synthesis

**Scheme 1:**
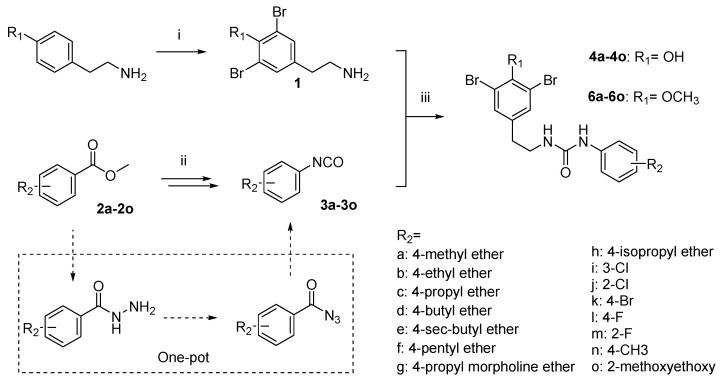
Synthesis of novel brominated tyrosine derivatives. Conditions: (**i**) 1.1 eq. Br_2_, AcOH, 80 °C, 39–51%; (**ii**) 5 eq, hydrazine hydrate 80%, 80 °C; 2 eq, NaNO_2_, HCl, 0 °C~80 °C, 17–31%; (**iii**) 1.05 eq. **3a**–**3o**, EDCI, HOBt, Et3N, DMF, rt., 52–57%.

The synthesis of brominated tyrosine derivatives followed the general reaction pathway outlined in Scheme 1. Tyramine is dissolved in acetic acid, and liquid bromine is added and reacted at 80 °C to obtain the hydrobromate of intermediate 1. Chemical synthesis method to access **3a**–**3o** was reported previously [8]; using **2a**–**2o** as starting materials, which were sequentially converted to the corresponding aryl hydrazides, followed by diazotization to afford aryl azides in approximately 40% yield. These aryl azide intermediates then underwent Curtius rearrangement in 1,2-dichloroethane (DCE) at 80 °C, generating substituted aromatic isocyanates with isolated yields of about 50%.

### 2.3. Inhibitory Activities of (-)-Itampolin A Skeleton Brominated Tyrosine Derivatives

The capacity of brominated tyrosine derivatives to inhibit p38α activity was evaluated using the ADP-Glo Kinase Assay. The evaluation results are summarized in Table 1. To comprehensively discuss structure–activity relationships (SARs), the activity of brominated tyrosine derivatives synthesized in our previous work is also summarized in Table 1. Compound 4l demonstrated potent p38α inhibition (IC_50_ = 9.7 ± 1.4 nM) with demethylation at fragment A, enhancing activity by 3- to 7-fold when paired with small-volume R2 substituents (*p* < 0.01, two-way ANOVA), suggesting synergistic steric optimization in the ATP-binding pocket; this translated to selective anti-proliferative efficacy against gefitinib-resistant PC9/GR cells. It also indicates that the absence of a methyl group enhances inhibitory activity against p38α. Furthermore, compounds **4k**–**4n** exhibit higher activity compared to **6k**–**6n**, suggesting that the methyl group’s absence exerts a more pronounced effect on enhancing activity when the R2 substituent has a smaller volume.

Compounds **4l**, **4m**, and **4o** demonstrated the most potent inhibitory activity against p38α, achieving IC_50_ values of 9.7 ± 1.4 nM, 10.2 ± 2.3 nM, and 11.3 ± 26.2 nM, respectively (Table 1). Notably, both **4l** and **4m** bear a fluorine atom, which likely originates from the ligand in the 4LOP complex (Supplementary Figure S1), thereby validating the BREED fragment-based drug design approach as a feasible strategy for kinase inhibitor development. The high activity of **4o** is attributed to its high structural similarity to **6o**, which we had investigated in our previous work. Based on the above results, compounds **4l** and **4m** were selected for further evaluation of anti-proliferative effects using the MTT assay in gefitinib-resistant NSCLC cells.

### 2.4. Evaluation of the Biological Activity of Compound **4l** Against Drug-Resistant NSCLC

**Figure 3:**
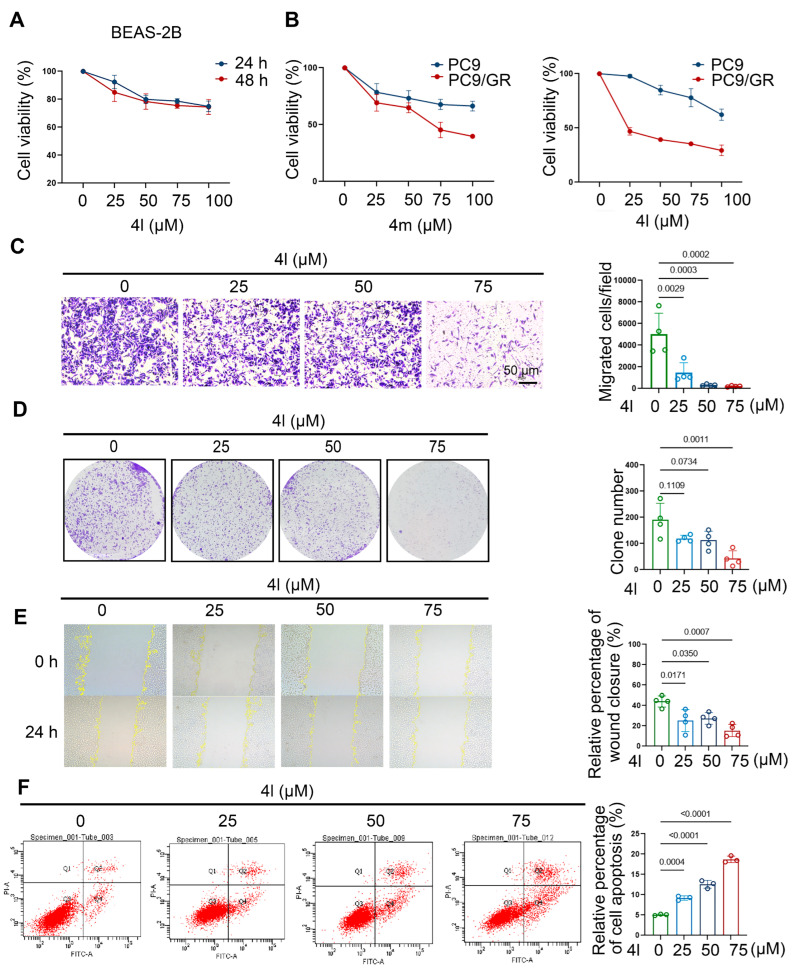
Effects of **4l** on various biological behaviors of PC9/GR cells. (**A**) The effect of MTT assay on the proliferation of BEAS-2B cells treated with different concentrations of **4l**. (**B**) The effect of MTT assay on the proliferation of gefitinib-sensitive (PC9) and resistant non-small cell lung cancer (PC9/GR) cells treated with different concentrations of **4m** and **4l**. The control group was 0.1% DMSO, and PC9 and PC9/GR cells were treated with **4m** and **4l** for 24 h. (**C**) Assessment of the migration ability of PC9/GR cells treated with different concentrations of **4l** using the Transwell method (*n* = 4). Scale bar = 50 μm. (**D**) Examination of the effect of compound **4l** on the clonogenic ability of PC9/GR cells using a colony formation assay (*n* = 4). (**E**) Analysis of the migration ability of PC9/GR cells treated with different concentrations of **4l** using a scratch assay (*n* = 4). (**F**) The detection of apoptosis in PC9/GR cells after treatment with different concentrations of **4l** using the FITC/PI staining method (*n* = 4). All graphs show means ± SDs. Statistical analysis was performed using One-way ANOVA or Two-way ANOVA and Tukey’s multiple comparisons test.

To evaluate the tumor-selective activity of compounds **4l** and **4m**, dose–response studies were performed on wild-type PC9 and gefitinib-resistant PC9/GR cell lines. Following 24-hour treatment with **4l** and **4m** (0–100 μM), a marked differential response was observed on **4l**: PC9 cells exhibited minimal growth inhibition (IC_50_ = 135.5 μM), whereas PC9/GR cells demonstrated significantly suppressed proliferation (IC_50_ = 35.2 μM), representing a nearly 4-fold increase in potency against the resistant phenotype (Figure 3B). While **4m** did not demonstrate significant selectivity towards cells with the drug-resistant phenotype (PC9: IC_50_ = 55.5 μM; PC9/GR: 39.0 μM). Based on these differential effects exhibited by **4l**, subsequent experiments were conducted using **4l**. Subtoxic concentrations of 25, 50, and 75 μM were employed to ensure specificity for drug-resistant phenotypes while preserving normal cell viability. Transwell migration assays demonstrated significant suppression of PC9/GR cell motility across all tested concentrations (Figure 3C). To assess long-term proliferative potential, clonogenic survival assays were performed. Cells pretreated with **4l** (24 h) were trypsinized to generate single-cell suspensions, plated at 300 cells/well, and cultured for 14 days. Crystal violet staining quantified colony formation, revealing statistically significant inhibition (*p* < 0.05) in **4l**-treated groups compared to untreated controls (Figure 3D). Cell migration ability was evaluated by the scratch healing assay. Consistent with these findings, ImageJ grayscale analysis revealed dose-dependent inhibition of migration, with relative wound healing areas decreasing from 44.08% (vehicle control) to 25.32% (25 μM), 27.45% (50 μM), and 15.45% (75 μM) (Figure 3E). All the above results indicate that **4l** can inhibit the invasion and migration of drug-resistant NSCLC cells. To evaluate the pro-apoptotic efficacy of **4l**, we employed a dual-staining approach using Annexin V-FITC and propidium iodide (PI) for quadrant-specific quantification of programmed cell death. Annexin V binds externalized phosphatidylserine (PS), a hallmark of early apoptosis, while PI permeabilization discriminates late-stage apoptotic or necrotic cells. Flow cytometric analysis of PC9/GR cells treated with **4l** (25–75 μM, 24 h) revealed a concentration-dependent increase in total apoptosis: 9.1% (25 μM), 13.13% (50 μM), and 19.53% (75 μM), all significantly elevated compared to the vehicle control (5.2%; *p* < 0.001) (Figure 3F).

The cytotoxicity of compound **4l** on normal human bronchial epithelial cells (BEAS-2B) was also evaluated using the MTT assay. Cells treated with 0.1% DMSO served as the vehicle control. Exposure to **4l** at concentrations ranging from 25 to 100 μM for 24 h showed no significant anti-proliferative effects on BEAS-2B cells. Even after prolonged treatment (48 h), cellular proliferation efficiency remained comparable to untreated controls (Figure 3A), confirming negligible cytotoxicity of **4l** at doses ≤ 100 μM in normal lung cells.

### 2.5. Screening of Target Genes for **4l** Antiresistant NSCLC Proliferation Based on Transcriptomics

**Figure 4:**
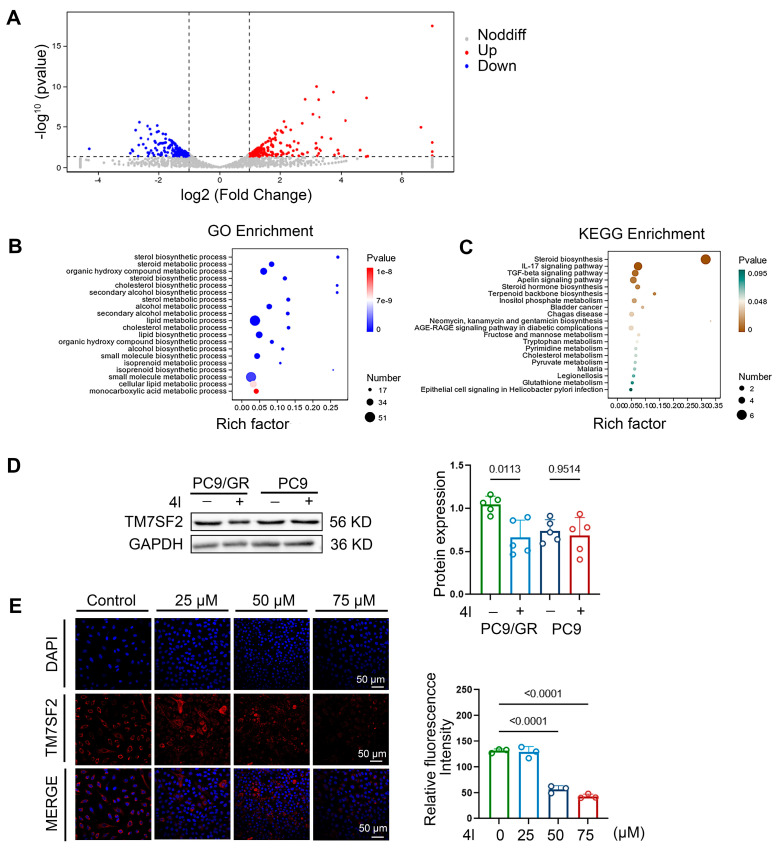
Screening of target genes for 4l antiresistant NSCLC proliferation based on transcriptomics. (**A**) The volcano plot shows the upregulated and downregulated genes in PC9/GR cells treated with compound **4l** compared to untreated PC9/GR cells. The significance threshold is set at *p* < 0.05, |Log2FoldChange| > 1. (**B**) The KEGG enrichment analysis results, where the number of enriched genes for each pathway is represented by the size of the dots; larger dots indicate a greater number of enriched genes. The pathways are ranked from high to low based on the *p*-value, which is also indicated by the color intensity; smaller P-values indicate higher confidence in the results. (**C**) Results of the GO enrichment analysis. (**D**) Western blot analysis examining the effect of compound **4l** on TM7SF2 protein expression (*n* = 5). (**E**) Cellular immunofluorescence assay of TM7SF2 level and colocalization in PC9-GR cells treated with **4l** for 24 h. Cell nuclei were stained with DAPI (scale bar = 50 μm, *n* = 3). All graphs show means  ±  SDs. Statistical analysis was performed using One-way ANOVA and Tukey’s multiple comparisons test.

To explore the mechanistic basis for the superior efficacy of compound **4l** in drug-resistant cells over wild-type cells, transcriptome sequencing studies were conducted. By comparing PC9/GR treated with the small molecule compound **4l** against untreated PC9/GR controls, we identified significant differentially expressed genes (DEGs). Subsequent Gene Ontology (GO) and Kyoto Encyclopedia of Genes and Genomes (KEGG) pathway enrichment analyses revealed key biological processes affected by **4l** treatment. The results of the KEGG enrichment analysis showed that most of these genes were enriched in the top five biological pathways, which include steroid biosynthesis, the pro-inflammatory cytokine interleukin 17 signaling pathway, the transforming growth factor beta-related signaling pathway that plays an important role in normal development and homeostasis, the Apelin signaling pathway that is crucial for angiogenesis, and steroid hormone biosynthesis (Figure 4A). The results of the GO enrichment analysis further emphasized the significant impact of the small molecule compound **4l** on steroid biosynthesis and metabolism in lung cancer cells, with the top five pathways being steroid biosynthesis, steroid metabolism, the metabolism of hydroxylated compounds, steroid biosynthesis, and cholesterol biosynthesis (Figure 4C). We further explored the enrichment analysis results of the downregulated genes and found that transmembrane 7 Superfamily Member 2 (TM7SF2), which encodes an enzyme involved in cholesterol metabolism, was significantly downregulated among the differentially expressed genes. TM7SF2 has been identified as a critical regulator of cholesterol biosynthesis and steroid metabolism in lung cancer cells, a finding central to overcoming chemoresistance in NSCLC [28]. Thus, we selected TM7SF2 as the target gene for further experiments and investigated the protein expression of TM7SF2 in **4l**-treated PC9/GR cells using immunoblotting. The experimental results indicated that the protein expression level of TM7SF2 was significantly reduced in **4l**-treated PC9/GR cells compared to untreated PC9/GR cells (Figure 4D,E).

### 2.6. Compound **4l** Reverses NSCLC Chemoresistance by TM7SF2 Mediated Modulating Cholesterol Homeostasis

**Figure 5:**
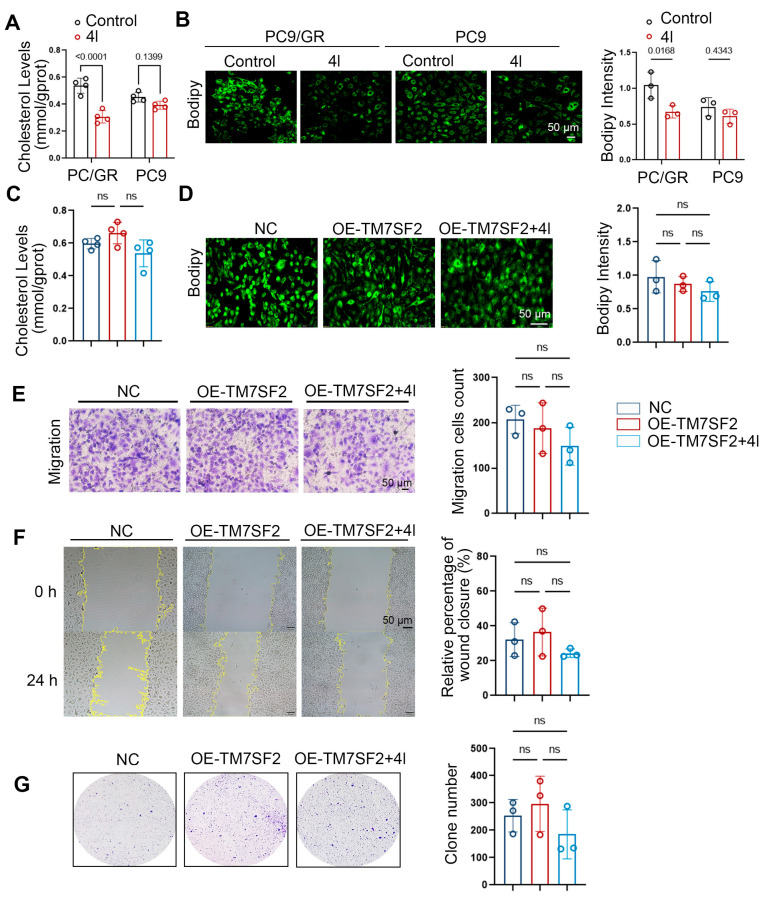
**4l** regulates intracellular cholesterol levels in a TM7SF2-dependent manner to combat NSCLC resistance. (**A**) Following treatment of the cells with **4l** for 24 h, the level of total cholesterol was detected (*n* = 4). (**B**) BODIPY staining was used to detect lipid droplet content in PC9/GR and PC9 cells by immunofluorescence. Scale bar = 50 µm. Green, BODIPY. Immunofluorescence intensity of BODIPY 493/503 in PC9/GR cells was measured (*n* = 3). (**C**) The cellular cholesterol levels of PC9/GR cells overexpressing TM7SF2 were assessed after treatment with **4l** (*n* = 3). (**D**) Representative images and quantification of transwell migration and matrigel invasion assays of PC9/GR cells treated with PPI (2 µM) and **4l** (*n* = 3). Scale bar = 50 μm. (**E**) The effect of **4l** on the migration ability of PC9/GR cells after overexpression of TM7SF2 (*n* = 3). Scale bar = 50 μm. (**F**) The effect of TM7SF2 overexpression on wound healing assay in PC9/GR cells (*n* = 3). Scale bar = 50 μm. (**G**) The effect of TM7SF2 overexpression on clonogenicity in PC9/GR cells (*n* = 3). Data presented as the mean ± SD. Statistical analysis was performed using One-way ANOVA or Two-way ANOVA and Tukey’s multiple comparisons test.

To investigate the relationship between intracellular cholesterol levels and gefitinib resistance, we measured cholesterol content in PC9 (gefitinib-sensitive) and PC9/GR (gefitinib-resistant) cells. Cholesterol quantification assays revealed significantly higher intracellular cholesterol levels in PC9/GR cells compared to PC9 cells. Treatment with compound **4l** reduced intracellular cholesterol levels in PC9/GR cells but had no significant effect on PC9 cells (Figure 5A). Furthermore, BODIPY 493/503 staining of lipid droplets (LDs) demonstrated increased LD accumulation in PC9/GR cells relative to PC9 cells. Compound **4l** treatment significantly inhibited this lipid accumulation in gefitinib-resistant PC9/GR cells (Figure 5B). To further elucidate the role of TM7SF2 in mediating the cholesterol-lowering effects of compound **4l** in PC9/GR cells, we generated a TM7SF2-overexpressing PC9/GR cell line via plasmid transfection. Results demonstrated that TM7SF2 overexpression abrogated the reduction in intracellular cholesterol levels induced by compound **4l** in PC9/GR cells (Figure 5C,D). Furthermore, TM7SF2 overexpression attenuated the inhibitory effects of compound **4l** on the migration, proliferation, and invasion of PC9/GR cells (Figure 5E–G). Collectively, these data indicate that compound **4l** primarily suppresses the malignant phenotype of gefitinib-resistant cells by inhibiting TM7SF2 expression.

### 2.7. **4l** Downregulates the Expression of TM7SF2 Through the Transcription Factor C/EBPβ

**Figure 6:**
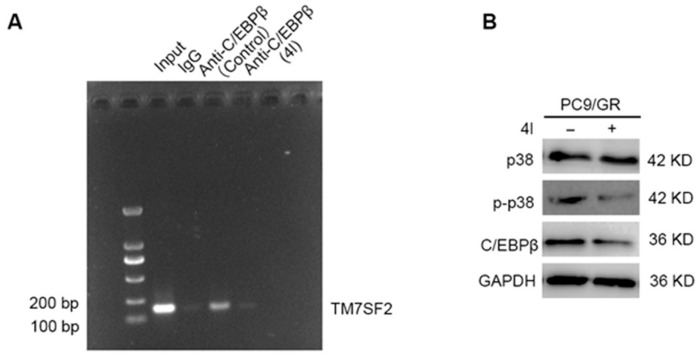
(**A**) Chromatin immunoprecipitation–PCR detection of C/EBPβ binding to the promoter region of the TM7SF2 gene. (**B**) Western blot analysis of the protein expression levels of p38, p-p38, and C/EBPβ in the cells.

Research shows that lipogenesis is a multi-stage process involving the expression of specific transcription factors from the CCAAT/enhancer-binding protein (C/EBP) and peroxisome proliferator-activated receptor (PPAR) families, requiring the coordination and continuous activation of multiple signaling events [29]. We obtained the gene promoter sequence of TM7SF2 from the NCBI database and utilized online databases JASPAR, UCSC, and hTFtarget to predict potential transcription factors for TM7SF2 (Table S2). By intersecting these results and focusing on the overlapping findings, we identified C/EBPβ. Some studies indicate that p38 can induce C/EBPβ expression through the activation of p38 MAPK, JNK, or ERK in different experimental environments [30,31]. We conducted Western blot experiments to detect changes in C/EBPβ expression and p38 protein activation and used ChIP and qPCR to determine whether C/EBPβ can target the TM7SF2 promoter region. The results showed that the Anti-C/EBPβ experimental group in the Input control and Control groups had specific amplification bands, while the negative control group and the Anti-C/EBPβ experimental group of PC9/GR cells treated with compound **4l** did not show specific amplification bands (Figure 6A,B). This indicates that C/EBPβ can indeed directly bind to the TM7SF2 promoter region (–582~−594 bp, GCCCCTGCTTGGA), and that compound **4l** can affect this binding, thereby influencing TM7SF2 protein expression. Furthermore, the strength of this direct binding may be related to intracellular cholesterol levels. Western blot results showed that **4l** can reduce C/EBPβ expression by inhibiting p38 activation, further downregulating TM7SF2 expression.

### 2.8. **4l** Significantly Inhibits Tumorigenicity of Drug-Resistant Strains In Vivo

**Figure 7:**
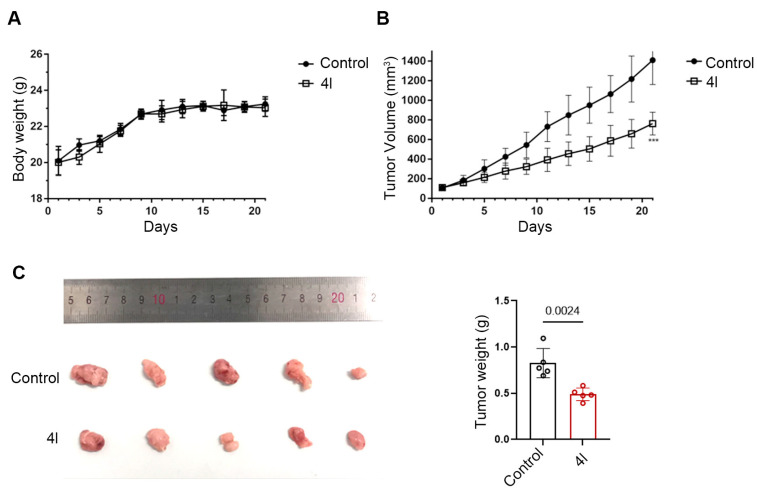
Tumor-bearing experiment results. (**A**) Weight changes of the two groups of nude mice over 21 days after treatment initiation (*n* = 5). (**B**) Changes in tumor volume in the body over 21 days after treatment initiation (*n* = 5). (**C**) Tumor tissue excised after the euthanasia of the nude mice (*n* = 5). Data presented as the mean ± SD. Statistical analysis was performed using Two-way ANOVA and Tukey’s multiple comparisons test. *** *p* < 0.001.

For many years, the technique of implanting human tumor cell lines into nude mice models has been widely used to predict the anti-cancer effects of targeted anti-cancer drugs. In the nude mouse tumor implantation experiment, we continuously monitored the changes in the mouse body weight after administration. The results showed that there was no significant decrease or fluctuation in body weight with the administration of compound **4l**, indicating that the dosage of compound **4l** used in this experiment did not have significant toxicity to the nude mice (Figure 7A). By administering 200 mg/kg of gefitinib and compound **4l** to the mice, we found that the administration of **4l** effectively inhibited the in vivo growth of PC9/GR nude mouse xenografts (*p* < 0.05). Compared to the control group (gefitinib-treated group), the **4l** treatment group showed a stronger inhibitory effect (Figure 7B). At the end of the experiment, after euthanizing the nude mice using the spinal dislocation method, we isolated the tumor tissues, weighed them, and conducted statistical analysis. The data obtained were consistent with the volume data recorded during the experimental process (Figure 7C).

## 3. Discussion

This study demonstrates that the marine-derived bromotyrosine derivative **4l** represents a promising chemotherapeutic agent for overcoming gefitinib resistance in NSCLC. By leveraging fragment-based drug design (FBDD) and structural optimization of the natural product itampolin A, we developed a simplified scaffold that selectively targets drug-resistant PC9/GR cells with minimal cytotoxicity to normal lung epithelia. The 12-fold higher potency of **4l** against PC9/GR versus parental PC9 cells underscores its potential as a tailored therapeutic for refractory lung cancer.

The mechanistic investigation revealed that **4l** disrupts cholesterol homeostasis, which is a critical vulnerability in resistant NSCLC [32,33,34,35,36]. Transcriptomic analysis identified TM7SF2, a key enzyme in cholesterol biosynthesis, as significantly downregulated upon **4l** treatment. This was corroborated by reduced intracellular cholesterol levels and lipid droplet accumulation in PC9/GR cells, effects abolished by TM7SF2 overexpression. These findings establish TM7SF2-mediated cholesterol metabolism as a druggable axis in chemoresistance, aligning with emerging evidence that cholesterol-rich lipid rafts facilitate survival signaling and drug efflux in resistant cancers. Furthermore, we elucidated the upstream regulatory mechanism: **4l** inhibits p38α MAPK activation, suppressing the transcription factor C/EBPβ. Chromatin immunoprecipitation confirmed C/EBPβ binding to the TM7SF2 promoter, an interaction disrupted by **4l**, thereby reducing TM7SF2 expression.

This p38α/C/EBPβ/TM7SF2 axis provides a novel molecular rationale for overcoming resistance, distinct from conventional ATP-competitive kinase inhibitors. Notably, compound **4l** exhibited robust performance in in vivo models, demonstrating significant suppression of PC9/GR xenograft growth without eliciting systemic toxicity. This favorable outcome may be attributed to the optimized pharmacokinetic properties inherited from the itampolin A scaffold, which enhances metabolic stability and bioavailability [37].

## 4. Materials and Methods

### 4.1. Drug Design

The three-dimensional structures of p38α in complex with protein binding partners were retrieved from the Protein Data Bank (PDB). We systematically selected all protein entries containing p38α chains and sorted them by “Total Number of Polymer Residues” to identify dimeric or multimeric assemblies (Supplementary Table S3). After removing redundant entries, eight unique PDB structures were curated for analysis (IDs: 6CAT, 2PUU, 4LOP, 5NZZ, 4TYH, 5ETA, 8A8M, 2ONL). These structures were then imported into the Protein Align/Superpose module of Molecular Operating Environment (MOE, version 2019.0102) software. Within the alignment workflow, p38α chains were designated as the reference blocks for structural superposition. The aligned conformations were subsequently processed through the BREED module to generate novel ligand scaffolds. Generated molecules were filtered using RO5 criteria to prioritize compounds with drug-like properties.

### 4.2. Chemistry

^1^H-NMR and ^13^C-NMR spectra were recorded on a Varian NMR spectrometer operating at 600 MHz for 1H and 150 MHz for ^13^C. All chemical shifts were measured in DMSO-d6 as solvents. All chemicals were purchased from Sinoreagent Chemical Reagent (Beijing, China) and were used as received, unless stated otherwise. Analytical TLC was performed on Haiyang (Qingdao Haiyang Chemical Co., Ltd., Qingdao, China) silica gel 60 F254 plates and visualized by UV and potassium permanganate staining. Flash column chromatography was performed on Haiyang (Qingdao Haiyang Chemical Co., Ltd.) gel 60 (40–63 mm). HPLC was performed on an Agilent 1260 Infinity II. The comprehensive characterization data and complete synthetic procedures of these novel derivatives were documented in the Supplementary Information.

### 4.3. Cell Culture and Reagents

The human normal lung epithelial cell line BEAS-2B, non-small cell lung carcinoma (NSCLC) cell line PC9, and gefitinib-resistant PC9/GR cell line were purchased from Procell Life Science & Technology Co., Ltd. (Wuhan, China). All cell lines were maintained in RPMI-1640 medium (BI, Beit Haemek, Mateh Asher, Israel) supplemented with 10% fetal bovine serum (FBS, Gibco, Thermo Fisher Scientific, Waltham, MA, USA) and 1% penicillin–streptomycin–amphotericin B sterile solution (Solarbio, Beijing, China). Cells were cultured at 37 °C in a humidified incubator with 5% CO_2_. For subculturing, cells were harvested using trypsin-EDTA solution upon reaching 70–80% confluence and resuspended at appropriate densities for subsequent experiments.

### 4.4. Cell Survival and Growth Assays

MTT: Cells (5 × 10^4^/mL) were treated with **4l** (25–100 μM, 24–48 h), incubated with MTT (10 μL/well, 4–6 h), and dissolved in DMSO. Absorbance (490 nm) was measured to assess viability.

Clonogenic Survival: Single-cell suspensions (300 cells/well) were cultured for 14 days. Colonies (>50 cells) were fixed (4% PFA), stained with crystal violet, and counted.

### 4.5. Cell Transfection and Plasmid Construction

The cDNA sequence encoding human TM7SF2 was synthesized by Youbio Biotechnology Co., Ltd. (Changsha, China) and cloned into the mammalian expression vector pcDNA3.1-3xFlag-C to generate N-terminal 3xFlag-tagged TM7SF2 (Flag-TM7SF2). For transient overexpression, PC9/GR cells were transfected with Flag-TM7SF2 or empty vector control using Lipofectamine™ reagent (Meilunbio, Dalian, China) at a DNA–Lipofectamine ratio of 1 μg–1 μL.

DNA–Lipofectamine complex formation: Diluted plasmid DNA and Lipofectamine reagent were separately mixed in Opti-MEM^®^ I Reduced Serum Medium (Gibco, USA), combined, and incubated at room temperature for 20 min. The DNA–Lipofectamine complexes were added dropwise to cells cultured in complete medium.

### 4.6. Wound Healing Assay

PC9/GR cells were grown to 90% confluence. A uniform wound was created in the monolayer using a sterile 200 µL pipette tip. The initial wound area was imaged immediately (0 h) using phase-contrast microscopy. After 24 h of incubation in complete medium, the wound area was re-imaged. The relative wound closure rate was calculated from the change in wound area using ImageJ software (version 1.53).

### 4.7. Cell Migration Assay

Cell migration was assessed using Transwell chambers (8.0 µm pore size; Corning, NY, USA). Cells suspended in serum-free medium were seeded into the upper chamber. The lower chamber contained 1 mL of DMEM supplemented with 10% FBS as a chemoattractant. Following 24 h of incubation, non-migrated cells on the upper membrane surface were removed. Migrated cells adherent to the lower membrane surface were fixed, stained with 0.1% crystal violet (Solarbio, China) for 15 min, and imaged. Migrated cells were quantified by counting five random fields per insert under a light microscope (Olympus, Tokyo, Japan).

### 4.8. Colony Formation Assay

PC9/GR cells were seeded into 6-well plates at a density of 800 cells per well and cultured in a 37 °C incubator for 2 weeks. The culture medium was then discarded, and the cells were washed with PBS. The cells were fixed with 4% paraformaldehyde for 30 min and stained with crystal violet (Solarbio, China) for 10 min. Finally, the number of cell colonies was counted.

### 4.9. Apoptosis Detection

Annexin V/PI Apoptosis: **4l** treated cells were stained with Annexin VFITC/PI (15 min, dark), analyzed by flow cytometry (Q2/Q3: late/early apoptosis).

### 4.10. Intracellular Cholesterol Quantification

Following sample collection, intracellular total cholesterol levels were quantified using a commercial Total Cholesterol (T-CHO) assay kit (Nanjing Jiancheng Bioengineering Institute, Nanjing, China) according to the manufacturer’s protocol. Cells were treated as indicated 24 h prior to analysis. Total cellular protein was extracted, and protein concentration was determined using a bicinchoninic acid (BCA) assay. Protein aliquots were loaded in duplicate into a 96-well microplate. Reagents were added sequentially as specified in the kit instructions. After incubation at 37 °C for 10 min, absorbance was measured at 510 nm using a microplate reader. Total cholesterol content was normalized to cellular protein concentration and expressed as μg cholesterol per mg protein.

### 4.11. Molecular Interaction and Expression Profiling

ChIP: **4l** treated cells were crosslinked (1% formaldehyde, 20 min), lysed, and sonicated. Chromatin was immunoprecipitated with target antibodies, washed, and eluted. DNA was purified for qPCR.

qPCR: SYBR Green-based reactions (20 μL) included primers (0.4 μM each) and template DNA.

Cycling: 95 °C (100 s); 40 cycles of 95 °C/15 s followed by 60 °C/60 s.

Western blot: Proteins extracted with RIPA buffer were quantified (BCA), separated by SDS-PAGE, transferred to PVDF membranes, and probed with antibodies. Signals were detected by ECL.

### 4.12. Tumor Growth and Response Monitoring

BALB/c nude mice (*n* = 12) were subcutaneously injected with PC9/GR cells (1 × 10^7^ cells/mouse). When tumors reached 50 mm^3^, mice received daily **4l** or vehicle (3 weeks). Tumor volume (0.52 × length × width^2^) and weight were recorded post-euthanasia.

### 4.13. Statistical Analysis

Data from ≥3 replicates were analyzed using GraphPad Prism v10.2.0 (*t*-test/ANOVA; *p* < 0.05, *p* < 0.01, *p* < 0.001). ImageJ quantified band intensities (Western blot) and migration/clonogenic data.

## 5. Conclusions

Leveraging insights from our previous studies on the scaffold of itampolin A, we designed and synthesized a series of previously unreported brominated tyrosine derivatives to facilitate the development of marine-derived drug resources. Among them, the efficacy of compound **4l** against gefitinib-resistant PC9/GR cells is 12 times that of wild-type PC9 cells, while its cytotoxicity to normal lung epithelium can be ignored. We confirmed that **4l** disrupts cholesterol homeostasis by inhibiting the p38/C/EBPβ signaling axis, preventing the binding of the transcription factor C/EBPβ to the *TM7SF2* gene and reducing its expression. Furthermore, **4l** significantly inhibited tumor growth in PC9/GR xenografts without inducing systemic toxicity, confirming its in vivo efficacy against chemotherapy-resistant tumors. Collectively, these findings establish TM7SF2-mediated cholesterol metabolism as a druggable vulnerability in refractory lung cancer and underscore marine-derived bromotyrosine scaffolds as promising chemical platforms for overcoming kinase inhibitor resistance.

## Figures and Tables

**Table 1:** p38α MAP kinase inhibitory activity of brominated tyrosine derivatives.

No.	IC_50_ (nM)	No.	IC_50_ (nM)
**4a**	265.5 ± 20.7	**6a** ^a^	262.7 ± 3.3
**4b**	170.0 ± 12.1	**6b**	221.4 ± 4.2
**4c**	296.6 ± 17.5	**6c**	701.4 ± 7.4
**4d**	914.5 ± 44.3	**6d**	-
**4e**	-	**6e**	920.3 ± 6.1
**4f**	-	**6f**	-
**4g**	167.4 ± 13.6	**6g**	11.7 ± 3.0
**4h**	849.5 ± 32.7	**6h**	329.4 ± 44.2
**4i**	13.9 ± 2.7	**6i**	17.5 ± 2.4
**4j**	74.4 ± 13.0	**6j**	71.2 ± 4.5
**4k**	51.1 ± 9.5	**6k**	169.0 ± 2.6
**4l**	9.7 ± 1.4	**6l**	21.5 ± 4.6
**4m**	11.3 ± 26.2	**6m**	13.6 ± 3.0
**4n**	38.7 ± 8.1	**6n**	299.6 ± 11.7
**4o**	10.2 ± 2.3	**6o**	7.9 ± 1.7
		BIRB-796	11.3 ± 0.2

^a^ Compounds **6a**–**6o** were synthesized in previous work.

## Data Availability

All data generated or analysed during this study are included in this published article and its Supplementary Information.

## Supplementary Materials

The following supporting information can be downloaded at https://www.mdpi.com/article/10.3390/md23090357/s1, Figure S1: The chemical structure of the ligands in the six protein-ligand complexes; Table S1: Generated molecules by BREED; Table S2: Promoter prediction of TM7SF2; Table S3: Rcsb_pdb_structure; Table S4: The original activity data of **4l** and **4m**.

## Abbreviations

The following abbreviations are used in this manuscript:

C/EBPβ	CCAAT-enhancer-binding proteins
GO	Gene Ontology
KEGG	Kyoto Encyclopedia of Gene and Genomes
NSCLC	Non-small cell lung cancer
