# Supplementary material for: Design and Synthesis of Marine-Inspired Itampolin A Derivatives to Overcome Chemoresistance in NSCLC via Cholesterol Homeostasis Modulation
Source: Mar Drugs. 2025 Sep 15;23(9):357. doi: 10.3390/md23090357 (PMC12471852; doi:10.3390/md23090357)
Supplement: Supplementary file 1 [file marinedrugs-23-00357-s001.zip › Supplementary information.docx]

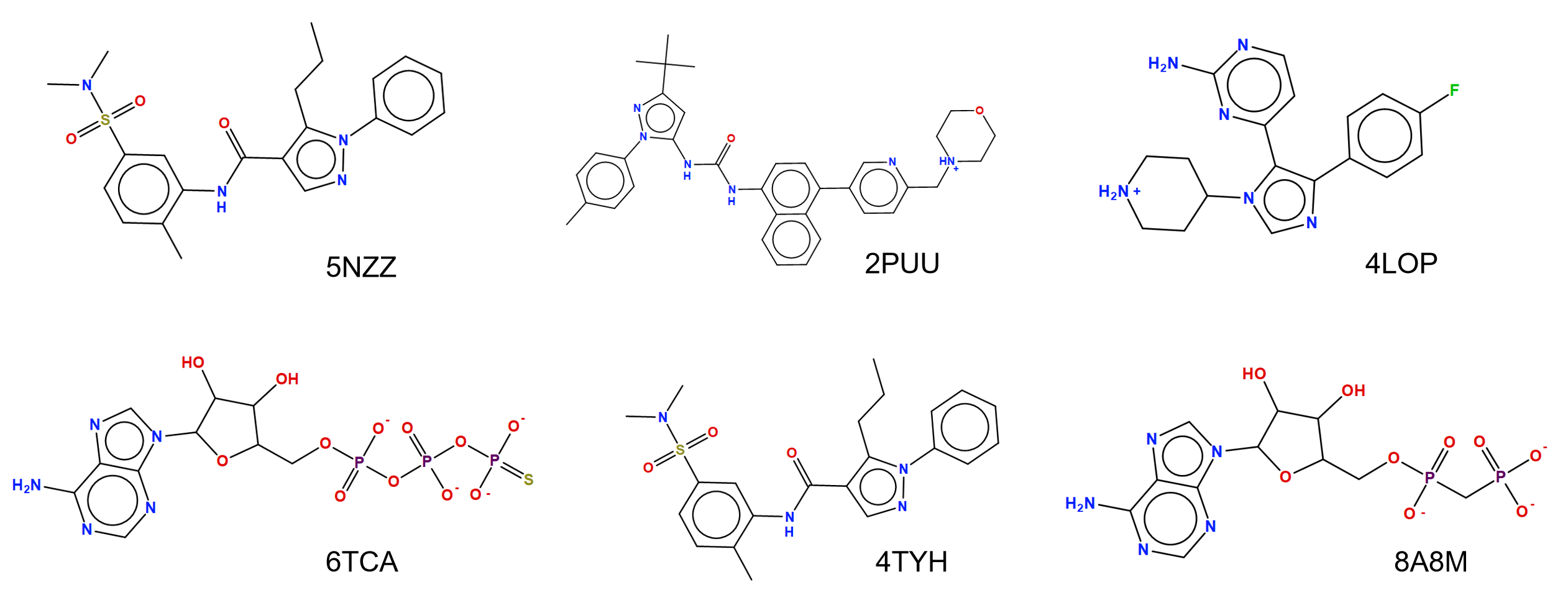


**Figure S1**. The chemical structure of the ligands in the six protein-ligand complexes

**1. Process for synthesis of the derivatives**

**4-(2-aminoethyl)-2,6-dibromophenol (1)**

The tyramine (15.00 g, 0.1 mol) was dissolved in glacial acetic acid, and 36.70 g (0.21 mol) of liquid bromine was added. The reaction was carried out at 70℃ for 1 hour, then cooled and filtered. The filter cake was rinsed with cold ethyl acetate: petroleum ether = 1:1 to obtain white solid powder, which was then dissolved in methanol. In a solvent with water =1:1, sodium bicarbonate solution was added dropwise until pH=7, and flocculent precipitate was formed. After filtration and drying, 18.71 g of white solid powder was obtained, with a yield of 58.5 %.

**Benzoyl azide derivatives (3a-3o)**

Ethyl 4-methoxybenzoate (**2a**) was dissolved in ethanol, treated with 5 equivalents of 80% hydrazine hydrate, and refluxed for 2 hours with reaction progress monitored by TLC; after cooling and standing, the resulting white needle-like crystals were collected by suction filtration. These crystals were then dissolved in a biphasic mixture of dichloromethane and water (DCM:H₂O = 1:4 *v/v*), slowly treated with 1.5 equivalents of concentrated hydrochloric acid, followed by the addition of 2 equivalents of sodium nitrite at 0 °C, and stirred for 15 minutes with reaction progress monitored by TLC; the aqueous layer was discarded and the organic layer concentrated under reduced pressure to afford a crude yellow oil, which was subsequently purified by silica gel column chromatography (eluent: ethyl acetate/petroleum ether = 1:5 *v/v*) to yield the product as a pale yellow oily liquid (**3a**). Compounds **3b-3o** were prepared according to the procedure described above, using the appropriate precursors **2b-2o** as the starting materials.

**1-(3,5-dibromo-4-hydroxyphenethyl)-3-(4-methoxyphenyl)urea (4a)**

4-(2-aminoethyl)-2,6-dibromophenol (**2**, 0.28 g, 0.95 mmol) was reacted with 4-methoxybenzoyl azide (**3a**, 0.17 g, 1 mmol) in 1, 2-dichloroethane at 80°C for 1 hour to obtain 0.31 g of white solid (**4a**), with a yield of 68.1 %, m.p.: 143-146°C. ^1^H NMR (600 MHz, DMSO-*d*_6_) δ 8.44 (s, 1 H), 8.19 (br. s., 1 H), 7.45 (s, 2 H), 7.39 (s, 2 H), 7.20 - 7.25 (m, *J*=8.5 Hz, 2 H), 6.71 - 6.85 (m, *J*=8.5 Hz, 2 H), 6.28 (d, *J*=8.5 Hz, 1 H), 4.32 - 4.48 (m, 1 H), 3.75 (s, 3 H), 3.68 (s, 3 H), 3.26 - 3.35 (m, 1 H), 3.20 (dd, *J*=12.1, 5.7 Hz, 1 H), 2.85 (dd, *J*=13.5, 4.0 Hz, 1 H), 2.69 - 2.78 (m, 1 H), 2.60 (d, *J*=4.9 Hz, 2 H). ^13^C NMR (150 MHz, DMSO-*d*_6_) δ 155.8, 154.3, 149.9, 149.7, 143.9, 141.5, 134.0, 132.7, 131.6. 120.3, 119.9, 119.8, 114.6, 114.2, 112.4, 55.5, 34.9, 34.5.

**1-(3,5-dibromo-4-hydroxyphenethyl)-3-(4-ethoxyphenyl)urea (4b)**

4-(2-aminoethyl)-2,6-dibromophenol (**2**, 0.28 g, 0.95 mmol) was reacted with 4-ethoxybenzoyl azide (**3b**, 0.19 g, 1 mmol) in 1, 2-dichloroethane at 80°C for 1 hour to obtain 0.35 g of white solid (**4b**), with a yield of 74.0%, m.p.: 196-203°C. ^1^H NMR (600 MHz, DMSO-*d*_6_) δ 8.29 (s, 1 H), 7.38 (s, 2 H), 7.12 - 7.28 (m, *J*=9.0 Hz, 2 H), 6.69 - 6.86 (m, *J*=9.0 Hz, 2 H), 6.06 (t, *J*=5.6 Hz, 1 H), 3.80 - 3.99 (m, 2 H), 3.27 (q, *J*=6.6 Hz, 2 H), 2.63 (t, *J*=7.0 Hz, 2 H), 1.29 (t, *J*=7.0 Hz, 3 H). ^13^C NMR (150 MHz, DMSO-*d*_6_) δ 155.8, 153.5, 150.3, 133.9, 132.6, 119.8, 114.8, 112.5, 63.4, 45.5, 34.5, 32.0, 15.1.

**1-(3,5-dibromo-4-hydroxyphenethyl)-3-(4-propoxyphenyl)urea (4c)**

4-(2-aminoethyl)-2,6-dibromophenol (**2**, 0.28 g, 0.95 mmol) was reacted with 4-propoxybenzoyl azide (**3c**, 0.20 g, 1 mmol) in 1, 2-dichloroethane at 80°C for 1 hour to obtain 0.23 g of white solid (**4c**), with a yield of 47.7%, m.p.: 203-209°C. ^1^H NMR (600 MHz, DMSO-*d*_6_) δ 9.69 (s, 1 H), 8.21 (s, 1 H), 7.41 (s, 2 H), 7.17 - 7.31 (m, 2 H), 6.74 - 6.85 (m, 2 H), 5.96 (t, *J*=5.6 Hz, 1 H), 3.84 (t, *J*=6.5 Hz, 2 H), 3.27 (q, *J*=6.8 Hz, 2 H), 2.64 (t, *J*=6.9 Hz, 2 H), 1.69 (s, *J*=7.0 Hz, 2 H), 0.96 (t, *J*=7.4 Hz, 3 H). ^13^C NMR (150 MHz, DMSO-*d*_6_) δ 155.7, 153.7, 149.3, 134.6, 133.9, 132.7, 119.8, 114.9, 112.3, 69.4, 34.5, 22.5, 10.8.

**1-(4-butoxyphenyl)-3-(3,5-dibromo-4- hydroxyphenethyl)urea (4d)**

4-(2-aminoethyl) -2,6-dibromophenol (**2**, 0.28 g, 0.95 mmol) was reacted with 4-butoxybenzoyl azide (**3d**, 0.21 g, 1 mmol) in 1, 2-dichloroethane at 80°C for 1 hour to obtain 0.19 g of white solid (**4d**), with a yield of 38.5 %, m.p.:178-182℃. ^1^H NMR (600 MHz, DMSO-*d*_6_) δ 8.21 (s, 1 H), 7.41 (s, 2 H), 7.16 - 7.29 (m, *J*=8.8 Hz, 2 H), 6.71 - 6.84 (m, *J*=8.8 Hz, 2 H), 5.96 (t, *J*=5.6 Hz, 1 H), 3.88 (t, *J*=6.5 Hz, 2 H), 3.27 (q, *J*=6.6 Hz, 3 H), 2.64 (t, *J*=6.9 Hz, 2 H), 1.57 - 1.75 (m, 2 H), 1.42 (sxt, *J*=7.5 Hz, 2 H), 0.92 (t, *J*=7.3 Hz, 3 H). ^13^C NMR (150 MHz, DMSO-*d*_6_) δ 155.7, 153.7, 149.3, 134.6, 133.9, 132.7, 119.8, 114.9, 112.3, 67.6, 34.5, 31.2, 19.1, 14.1.

**1-(4-(sec-butoxy)phenyl)-3-(3,5-dibromo-4- hydroxyphenethyl)urea (4e)**

4-(2-aminoethyl)-2,6-dibromophenol (**2**, 0.28 g, 0.95 mmol) was dissolved in 1,2-dichloroethane, then 4-(sec-butoxy)benzoyl azide (**3e**, 0.21 g, 1 mmol) was added and reacted at 80 ℃ for 1 hour to give 0.19 g of white solid (**4e**). The yield is 63.3 %, m.p.:161-165 ℃. ^1^H NMR (600 MHz, DMSO-*d*_6_) δ 8.21 (s, 1 H), 7.40 (s, 2 H), 7.13 - 7.29 (m, 2 H), 6.73 - 6.85 (m, 2 H), 5.97 (t, *J*=5.6 Hz, 1 H), 4.24 (sxt, *J*=6.0 Hz, 1 H), 3.27 (q, *J*=6.8 Hz, 2 H), 2.59 - 2.70 (m, 2 H), 1.48 - 1.67 (m, 2 H), 1.18 (d, *J*=6.0 Hz, 3 H), 0.90 (t, *J*=7.4 Hz, 3 H). ^13^C NMR (150 MHz, DMSO-*d*_6_) δ 155.6, 140.9, 138.0, 132.2, 128.8, 122.0, 118.6, 115.2, 113.2, 67.9, 34.7, 28.9, 28.1, 22.3, 14.3.

**1-(3,5-dibromo-4- hydroxyphenethyl)-3-(4-(pentyloxy)phenyl)urea (4f)**

4-(2-aminoethyl)-2,6-dibromophenol (**2**, 0.28 g, 0.95 mmol) was dissolved in 1,2-dichloroethane, then 4-(pentyloxy)benzoyl azide (**3f**, 0.23 g, 1 mmol) was added and reacted at 80 ℃ for 1 hour to give 0.28 g of white solid (**4f**). The yield is 54.7 %, m.p.:163-168 ℃. ^1^H NMR (600 MHz, DMSO-*d*_6_) δ 8.26 (s, 1 H), 7.35 - 7.44 (m, 2 H), 7.15 - 7.30 (m, *J*=9.0 Hz, 2 H), 6.73 - 6.88 (m, *J*=9.0 Hz, 2 H), 6.03 (t, *J*=5.6 Hz, 1 H), 3.80 - 3.96 (m, 2 H), 3.26 (q, *J*=6.7 Hz, 2 H), 2.63 (t, *J*=6.9 Hz, 2 H), 1.61 - 1.78 (m, 2 H), 1.29 - 1.41 (m, 4 H), 0.89 (t, *J*=7.2 Hz, 3 H). ^13^C NMR (150 MHz, DMSO-*d*_6_) δ 155.8, 153.7, 133.9, 132.6, 119.8, 114.9, 112.5, 67.9, 34.52, 28.9, 28.1, 22.3, 14.3.

**1-(3,5-dibromo-4- hydroxyphenethyl)-3-(4-(3-morpholinopropoxy)phenyl)urea (4g)**

4-(2-aminoethyl)-2,6-dibromophenol (**2**, 0.28 g, 0.95 mmol) was dissolved in 1,2-dichloroethane, then 4-(3-morpholinopropoxy)benzoyl azide (**3g**, 0.29 g, 1 mmol) was added and reacted at 80 ℃ for 1 hour to give 0.23 g of white solid (**4g**). The yield is 41.2 %, m.p.:175-180℃. ^1^H NMR (600 MHz, DMSO-*d*_6_) δ 9.79 (br. s., 1 H), 8.29 (s, 1 H), 7.41 (s, 2 H), 7.19 - 7.31 (m, *J*=8.8 Hz, 2 H), 6.74 - 6.85 (m, *J*=8.8 Hz, 2 H), 6.02 (t, *J*=5.5 Hz, 1 H), 3.94 (t, *J*=6.1 Hz, 2 H), 3.63 (br. s., 4 H), 3.27 (q, *J*=6.6 Hz, 2 H), 2.65 (t, *J*=6.9 Hz, 2 H), 1.90 (br. s., 2 H). ^13^C NMR (150 MHz, DMSO-*d*_6_) δ 155.7, 153.5, 149.3, 134.6, 132.7, 119.8, 114.9, 112.3, 66.0, 34.5, 31.2, 22.5, 10.8.

**1-(3,5-dibromo-4-hydroxyphenethyl)-3-(4-isopropoxyphenyl)urea (4h)**

4-(2-aminoethyl)-2,6-dibromophenol (**2**, 0.28 g, 0.95 mmol) was dissolved in 1,2-dichloroethane, then 4-isopropoxybenzoyl azide (**3h**, 0.20 g, 1 mmol) was added and reacted at 80 ℃ for 1 hour to give 0.17 g of white solid (**4h**). The yield is 34.7 %, m.p.:185-190℃. ^1^H NMR (600 MHz, DMSO-*d*_6_) δ 9.58 (br. s., 1 H), 8.22 (s, 1 H), 7.40 (s, 2 H), 7.14 - 7.31 (m, 2 H), 6.70 - 6.87 (m, 2 H), 5.98 (t, *J*=5.6 Hz, 1 H), 4.47 (dt, *J*=12.0, 6.0 Hz, 1 H), 3.28 (q, *J*=6.8 Hz, 2 H), 2.58 - 2.70 (m, 2 H), 1.22(d, *J*=6.0 Hz, 6 H). ^13^C NMR (150 MHz, DMSO-*d*_6_) δ 155.7, 153.7, 133.8, 133.4, 119.9, 117.6, 114.9, 69.4, 34.7, 22.5, 10.8.

**1-(3-chlorophenyl)-3-(3,5-dibromo-4-hydroxyphenethyl)urea (4i)**

4-(2-aminoethyl)-2,6-dibromophenol (**2**, 0.28 g, 0.95 mmol) was dissolved in 1,2-dichloroethane, then 3-chlorobenzoyl azide (**3i**, 0.18 g, 1 mmol) was added and reacted at 80 ℃ for 1 hour to give 0.29 g of white solid (**4i**). The yield is 67.3 %, m.p.:168-173℃. ^1^H NMR (600 MHz, DMSO-*d*_6_) δ 8.53 (s, 1 H), 7.25 - 7.32 (m, 2 H), 7.23 (s, 1 H), 7.16 (d, *J*=8.1 Hz, 1 H), 7.07 (t, *J*=7.8 Hz, 1 H), 6.69 (d, *J*=7.5 Hz, 1 H), 6.27 (t, *J*=5.4 Hz, 1 H), 3.24 (s, 2 H), 2.59 (s, 2 H). ^13^C NMR (150 MHz, DMSO-*d*_6_) δ 155.6, 140.9, 138.0, 132.2, 128.8, 122.0, 118.6, 115.2, 113.2, 34.6, 21.6.

**1-(2-chlorophenyl)-3-(3,5-dibromo-4-hydroxyphenethyl)urea (4j)**

4-(2-aminoethyl)-2,6-dibromophenol (**2**, 0.28 g, 0.95 mmol) was dissolved in 1,2-dichloroethane, then 2-chlorobenzoyl azide (**3j**, 0.18 g, 1 mmol) was added and reacted at 80 ℃ for 1 hour to give 0.22 g of white solid (**4j**). The yield is 51.8 %, m.p.:221-225℃. ^1^H NMR (600 MHz, DMSO-*d*_6_) δ 8.00 (s, 1 H), 7.43 (s, 2 H), 7.38 (dd, *J*=8.0, 1.4 Hz, 1 H), 7.20 - 7.26 (m, 1 H), 6.87 - 7.07 (m, 2 H), 3.30 - 3.34 (m, 2 H), 2.67 (t, *J*=6.9 Hz, 2 H). ^13^C NMR (150 MHz, DMSO-*d*_6_) δ 155.7, 153.7, 149.3, 132.7, 119.8, 114.9, 112.3, 34.5, 22.5.

**1-(4-bromophenyl)-3-(3,5-dibromo-4-hydroxyphenethyl)urea (4k)**

4-(2-aminoethyl)-2,6-dibromophenol (**2**, 0.28 g, 0.95 mmol) was dissolved in 1,2-dichloroethane, then 4-bromobenzoyl azide (**3k**, 0.23 g, 1 mmol) was added and reacted at 80 ℃ for 1 hour to give 0.21 g of white solid (**4k**). The yield is 44.1 %, m.p.:210-215℃. ^1^H NMR (600 MHz, DMSO-*d*_6_) δ 8.44 (s, 1 H), 8.19 (br. s., 1 H), 7.45 (s, 2 H), 7.39 (s, 2 H), 7.20 - 7.25 (m, *J*=8.5 Hz, 2 H), 6.71 - 6.85 (m, *J*=8.5 Hz, 2 H), 6.28 (d, *J*=8.5 Hz, 1 H), 4.32 - 4.48 (m, 1 H), 3.75 (s, 3 H), 3.68 (s, 3 H), 3.26 - 3.35 (m, 1 H), 3.20 (dd, *J*=12.1, 5.7 Hz, 1 H), 2.85 (dd, *J*=13.5, 4.0 Hz, 1 H), 2.69 - 2.78 (m, 1 H), 2.60 ppm (d, *J*=4.9 Hz, 2 H). ^13^C NMR (150 MHz, DMSO-*d*_6_) δ 155.7, 133.4, 119.6, 117.6, 114.9, 34.7, 22.5.

**1-(3,5-dibromo-4-hydroxyphenethyl)-3-(4-fluorophenyl)urea (4l)**

4-(2-aminoethyl)-2,6-dibromophenol (**2**, 0.28 g, 0.95 mmol) was dissolved in 1,2-dichloroethane, then 4-fluorobenzoyl azide (**3l**, 0.16 g, 1 mmol) was added and reacted at 80 ℃ for 1 hour to give 0.15 g of white solid (**4l**). The yield is 36.7 %, m.p.:179-186℃. ^1^H NMR (600 MHz, DMSO-*d*_6_) δ 8.48 (s, 1 H), 7.39 - 7.44 (m, 2 H), 7.34 - 7.39 (m, 2 H), 6.93 - 7.13 (m, 2 H), 6.08 (t, *J*=5.6 Hz, 1 H), 3.29 (q, *J*=6.8 Hz, 2 H), 2.65 ppm (t, *J*=7.0 Hz, 2 H). ^13^C NMR (150 MHz, DMSO-*d*_6_) δ 155.6, 149.5, 137.2, 132.7, 119.6, 115.5, 115.4, 112.3, 34.4.

**1-(3,5-dibromo-4-hydroxyphenethyl)-3-(2-fluorophenyl)urea (4m)**

4-(2-aminoethyl)-2,6-dibromophenol (**2**, 0.28 g, 0.95 mmol) was dissolved in 1,2-dichloroethane, then 2-fluorobenzoyl azide (**3l**, 0.16 g, 1 mmol) was added and reacted at 80 ℃ for 1 hour to give 0.19 g of white solid (**4l**). The yield is 45.3 %, m.p.:175-179℃. ^1^H NMR (600 MHz, DMSO-*d*_6_) δ 8.44 (s, 1 H), 8.19 (br. s., 1 H), 7.45 (s, 2 H), 7.39 (s, 2 H), 7.20 - 7.25 (m, *J*=8.5 Hz, 2 H), 6.71 - 6.85 (m, *J*=8.5 Hz, 2 H), 6.28 (d, *J*=8.5 Hz, 1 H), 4.32 - 4.48 (m, 1 H), 3.75 (s, 3 H), 3.68 (s, 3 H), 3.26 - 3.35 (m, 1 H), 3.20 (dd, *J*=12.1, 5.7 Hz, 1 H), 2.85 (dd, *J*=13.5, 4.0 Hz, 1 H), 2.69 - 2.78 (m, 1 H), 2.60 (d, *J*=4.9 Hz, 2 H). ^13^C NMR (150 MHz, DMSO-*d*_6_) δ 155.2, 152.0, 139.6, 133.4, 128.6, 124.7, 117.6, 115.2, 60.7, 34.6.

**1-(3,5-dibromo-4-hydroxyphenethyl)-3-(p-tolyl)urea (4n)**

4-(2-aminoethyl)-2,6-dibromophenol (**2**, 0.28 g, 0.95 mmol) was dissolved in 1,2-dichloroethane, then 4-methylbenzoyl azide (**3n**, 0.16 g, 1 mmol) was added and reacted at 80 ℃ for 1 hour to give 0.11 g of light yellow solid (**4n**). The yield is 27.4 %,m.p.:178-183℃. ^1^H NMR (600 MHz, DMSO-*d*_6_) δ 9.06 (br. s., 1 H), 8.35 (s, 1 H), 7.34 - 7.47 (m, 2 H), 7.17 - 7.29 (m, *J*=8.5 Hz, 2 H), 6.93 - 7.06 (m, *J*=8.3 Hz, 2 H), 6.07 (t, *J*=5.6 Hz, 1 H), 3.27 (q, *J*=6.8 Hz, 2 H), 2.63 (t, *J*=7.0 Hz, 2 H), 2.20 (s, 3 H). ^13^C NMR (150 MHz, DMSO-*d*_6_) δ 155.6, 138.3, 132.9, 129.4, 118.1, 112.5, 34.5, 20.7.

**1-(3,5-dibromo-4-hydroxyphenethyl)-3-(4-(2-methoxyethoxy)phenyl)urea (4o)**

4-(2-aminoethyl)-2,6-dibromophenol (**2**, 0.28 g, 0.95 mmol) was dissolved in 1,2-dichloroethane, then 4-(2-methoxyethoxy)benzoyl azide (**3o**, 0.22 g, 1 mmol) was added and reacted at 80 ℃ for 1 hour to give 0.18 g of off-white solid (**4o**). The yield is 38.2 %, m.p.:195-199℃. ^1^H NMR (600 MHz, DMSO-*d*_6_) δ 9.54 (br. s., 1 H), 8.24 (s, 1 H), 7.40 (s, 2 H), 7.16 - 7.29 (m, 2 H), 6.73 - 6.88 (m, 2 H), 5.99 (t, *J*=5.7 Hz, 1 H), 3.96 - 4.11 (m, 2 H), 3.51 - 3.69 (m, 2 H), 3.29 (s, 3 H), 3.25 - 3.29 (m, 2 H), 2.64 (t, *J*=6.9 Hz, 2 H). ^13^C NMR (150 MHz, DMSO-*d*_6_) δ 155.7, 153.5, 132.7, 119.8, 114.9, 112.3, 70.9, 67.4, 58.5, 40.7, 34.5.

**2. spectrum of the derivatives**

**
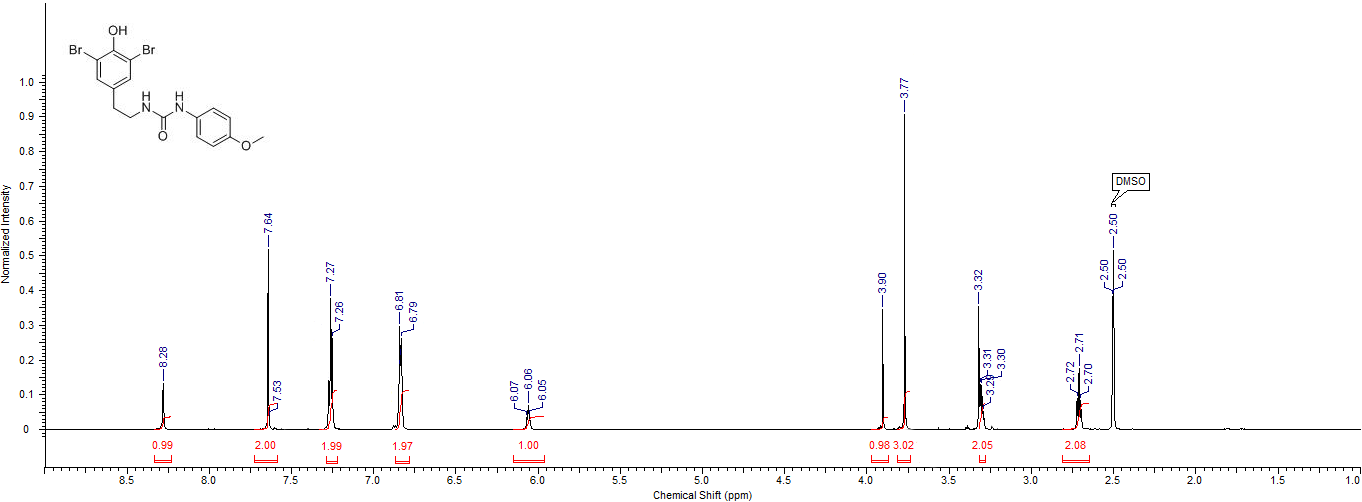
**

**4a ^1^H-NMR**

**
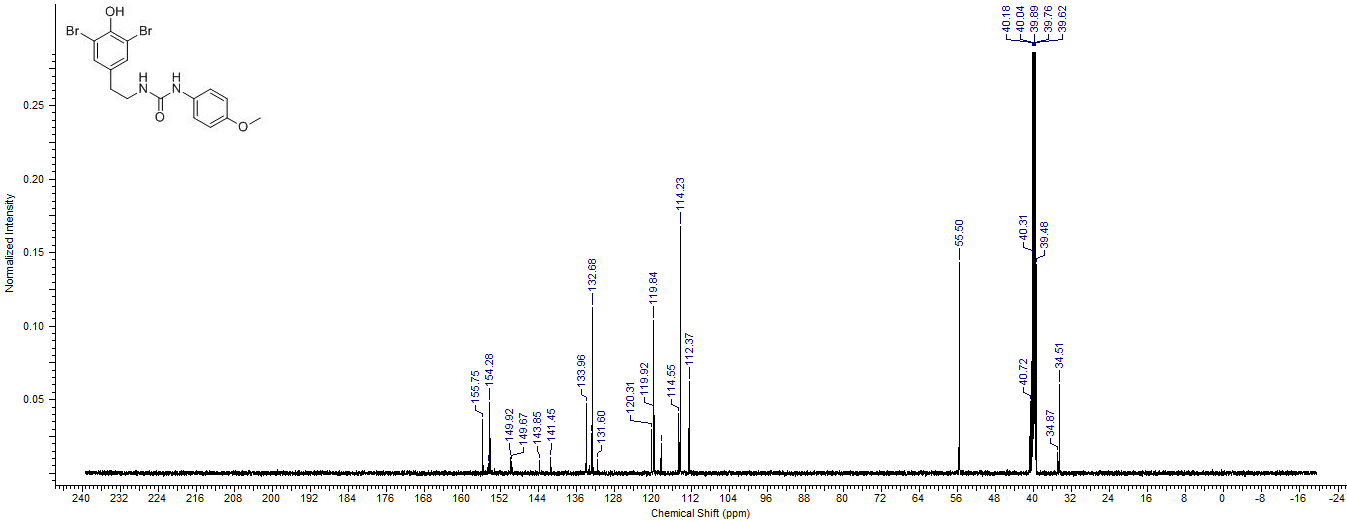
**

**4a ^13^C-NMR**

**
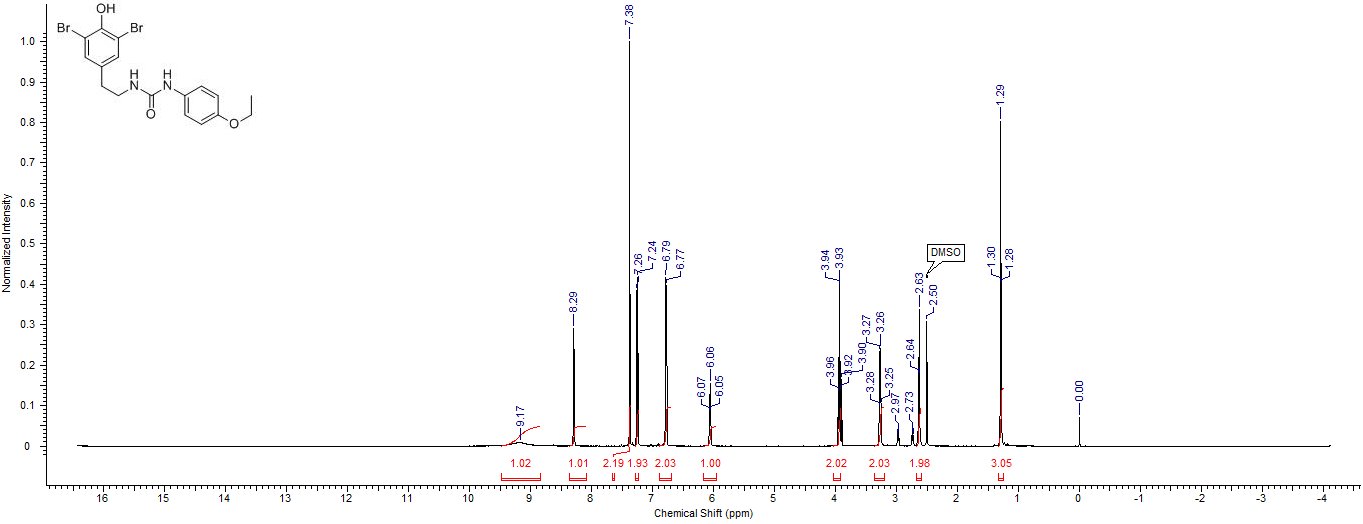
**

**4b ^1^H-NMR**

**
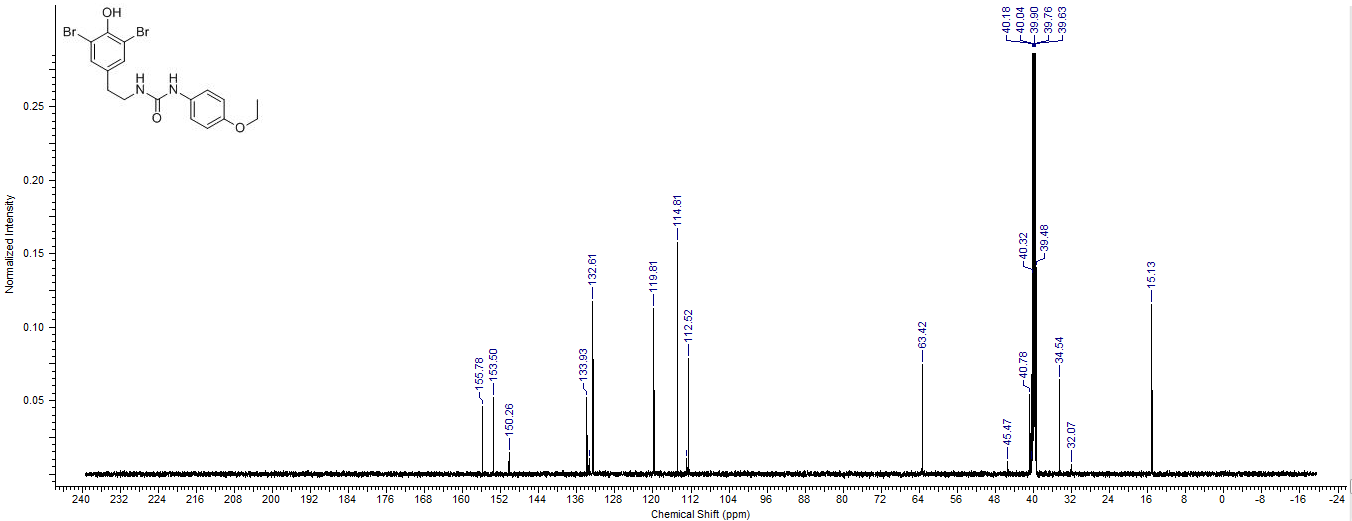
**

**4b ^13^C-NMR**

**
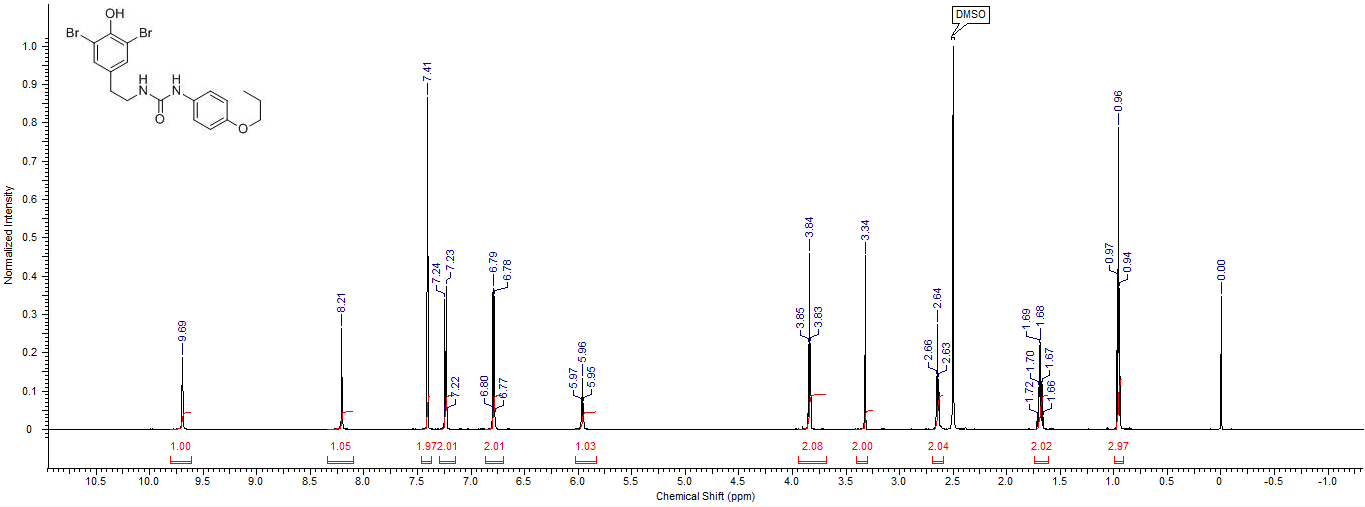
**

**4c ^1^H-NMR**

**
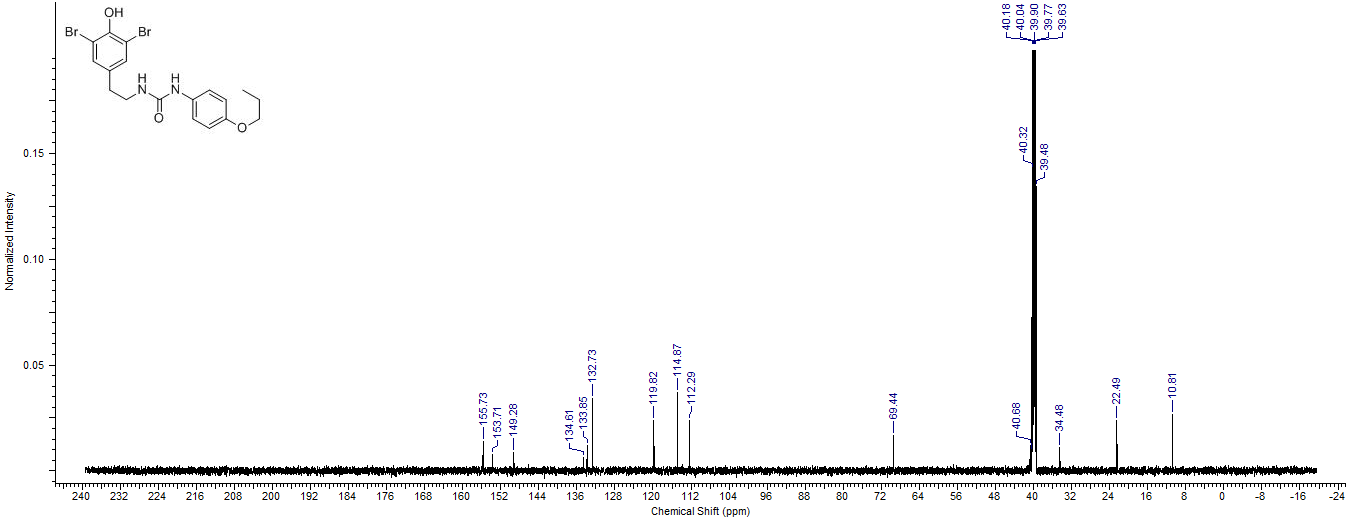
**

**4c ^13^C-NMR**

**
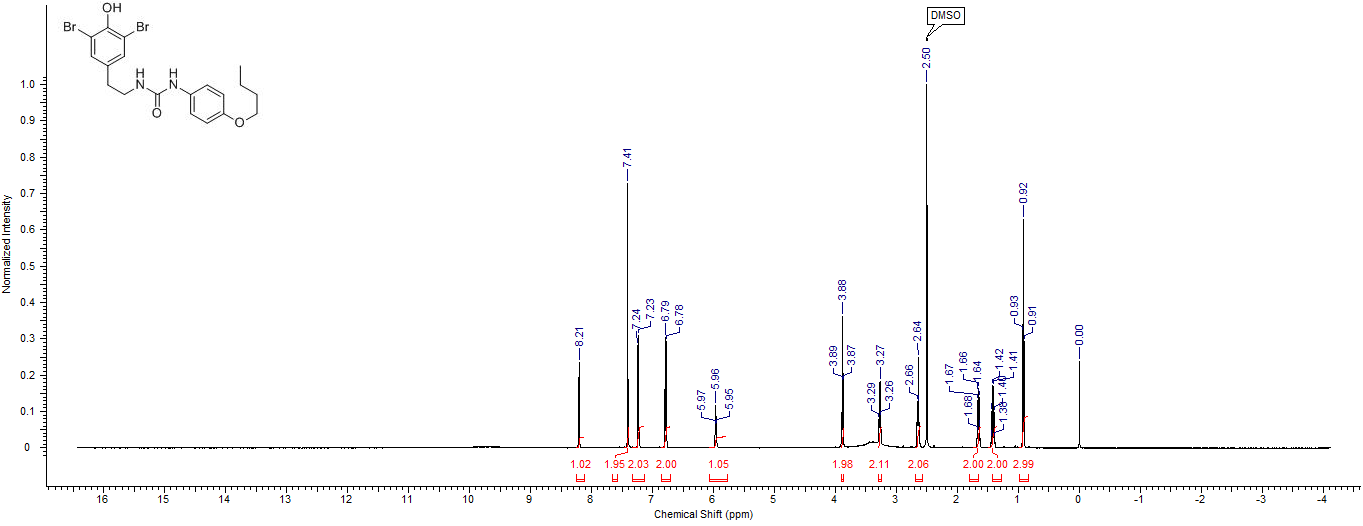
**

**4d ^1^H-NMR**

**
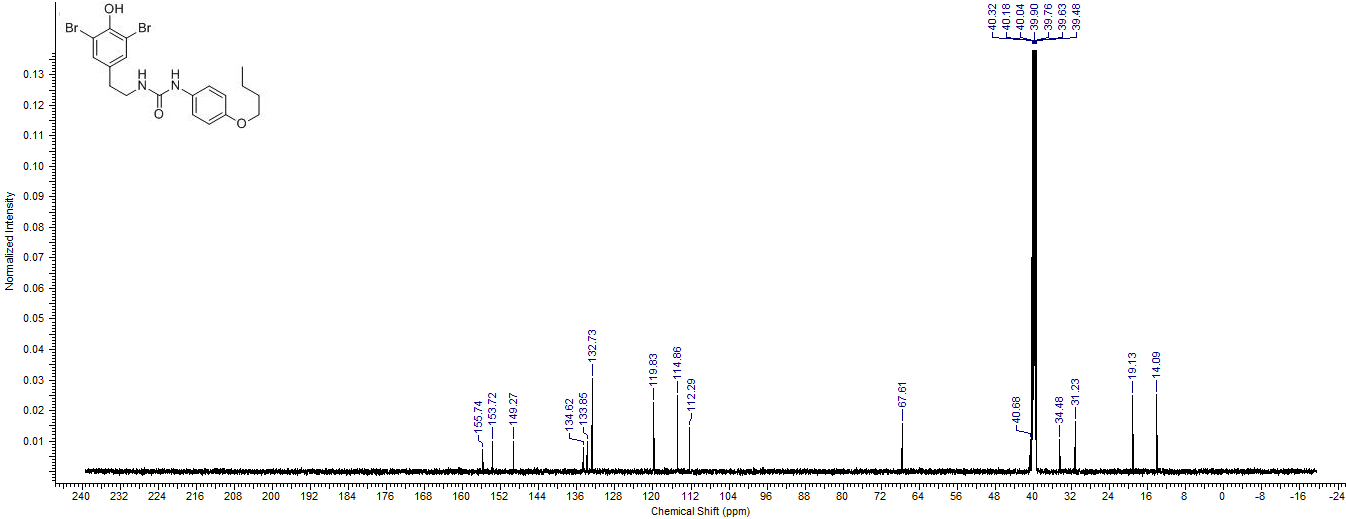
**

**4d ^13^C-NMR**

**
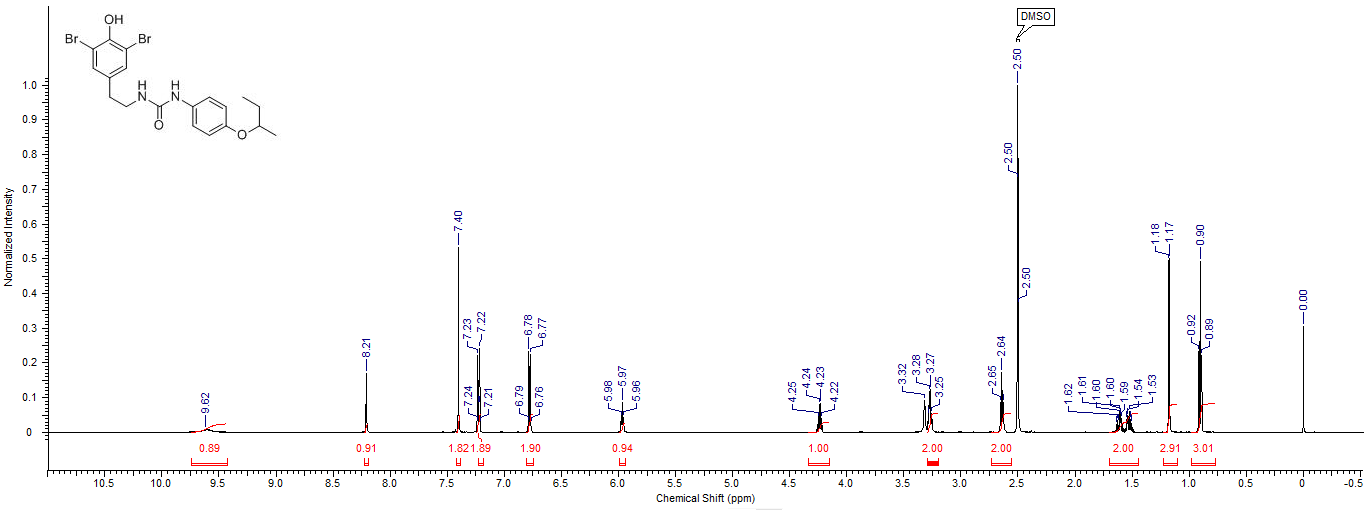
**

**4e ^1^H-NMR**

**
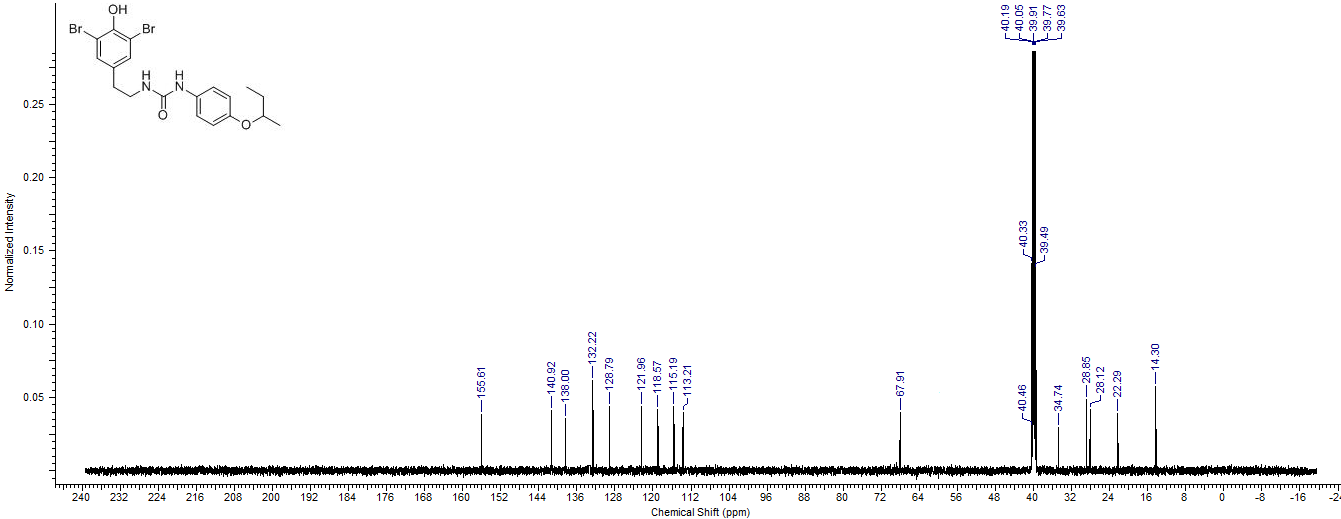
**

**4e ^13^C-NMR**

**
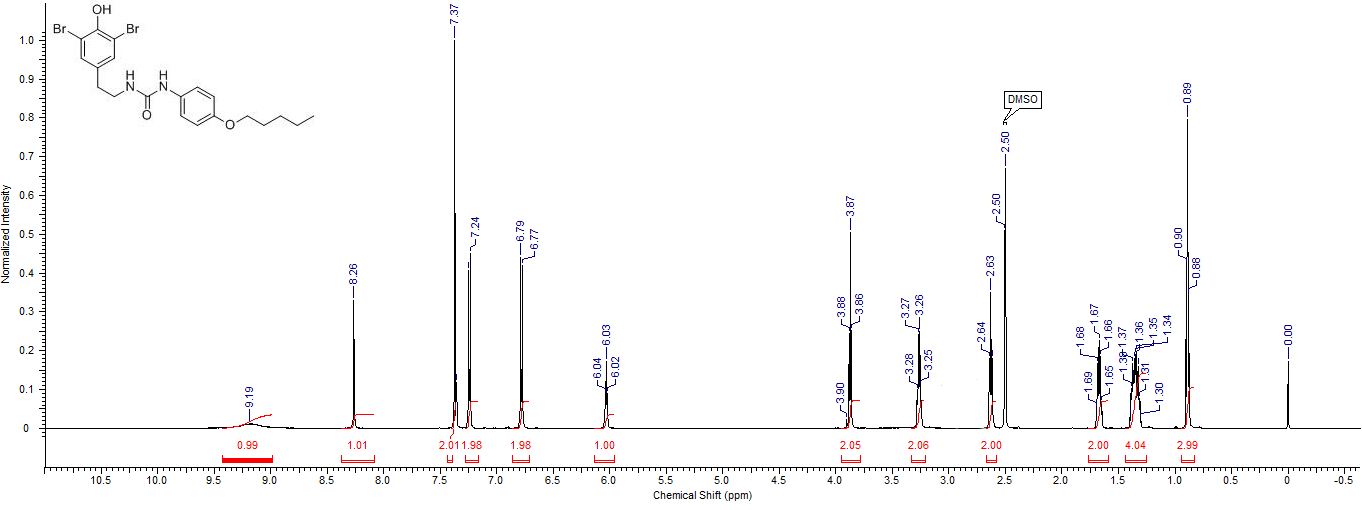
**

**4f ^1^H-NMR**

**
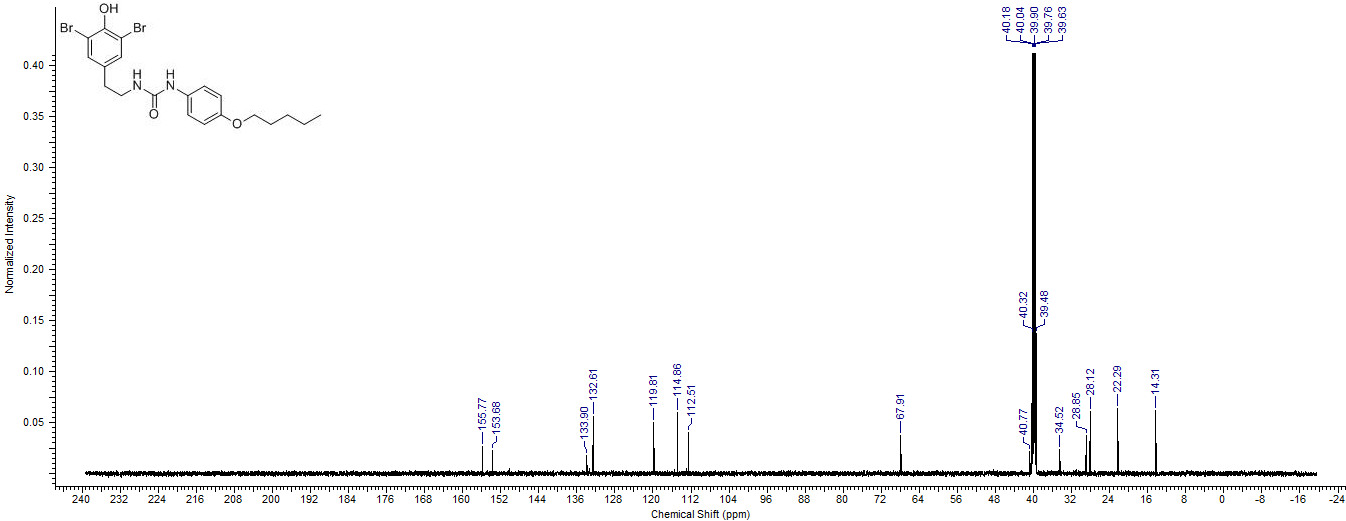
**

**4f ^13^C-NMR**

**
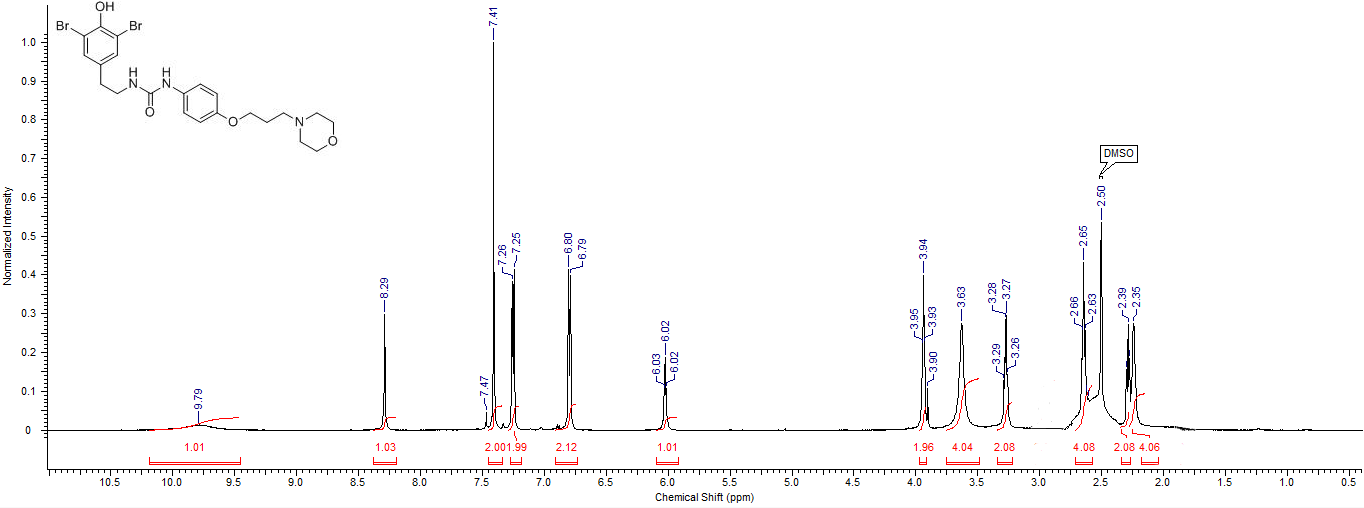
**

**4g ^1^H-NMR**

**
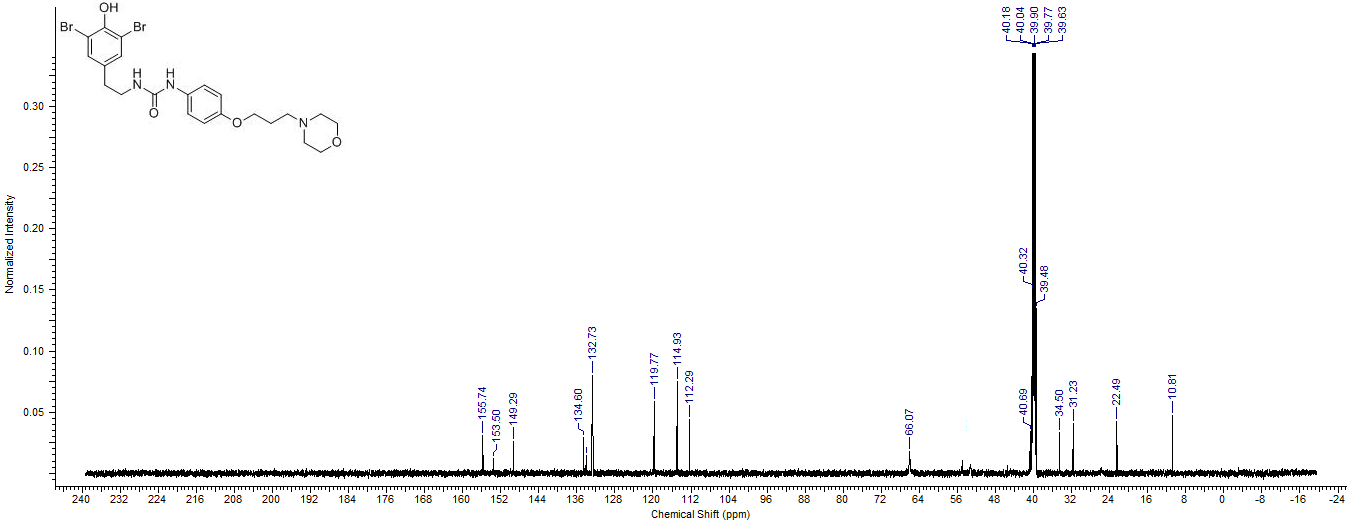
**

**4g ^13^C-NMR**

**
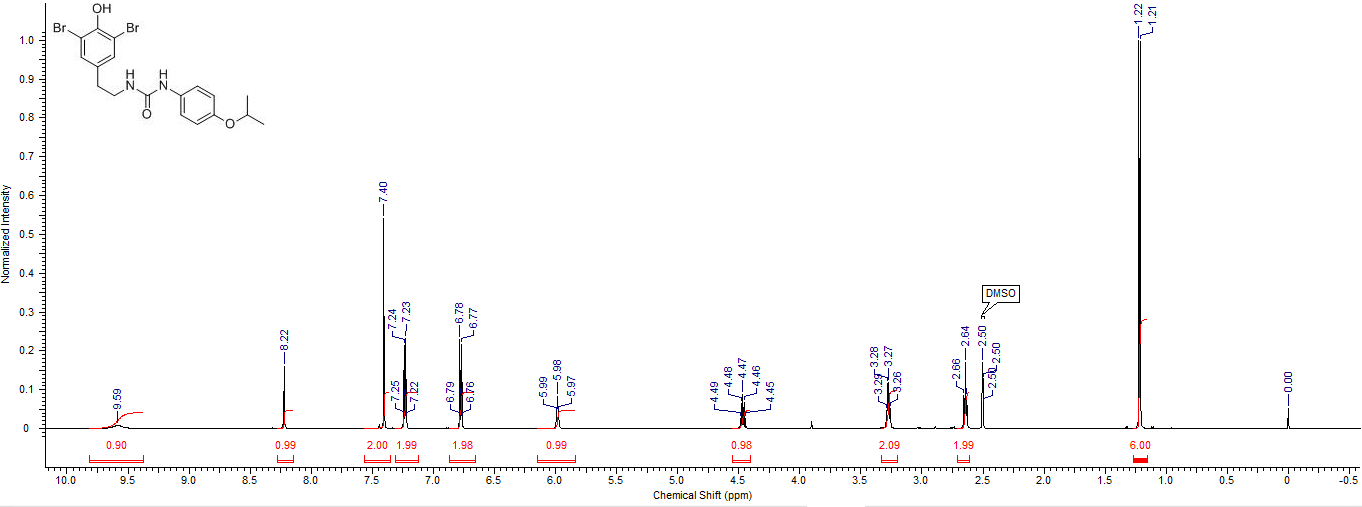
**

**4h ^1^H-NMR**

**
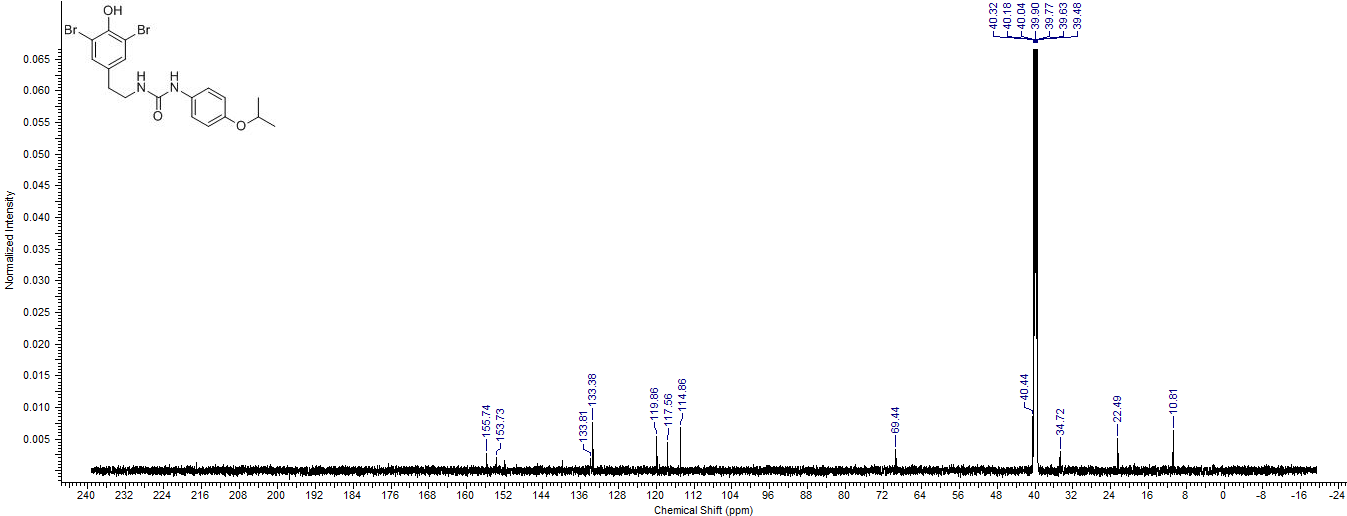
**

**4h ^13^C-NMR**

**
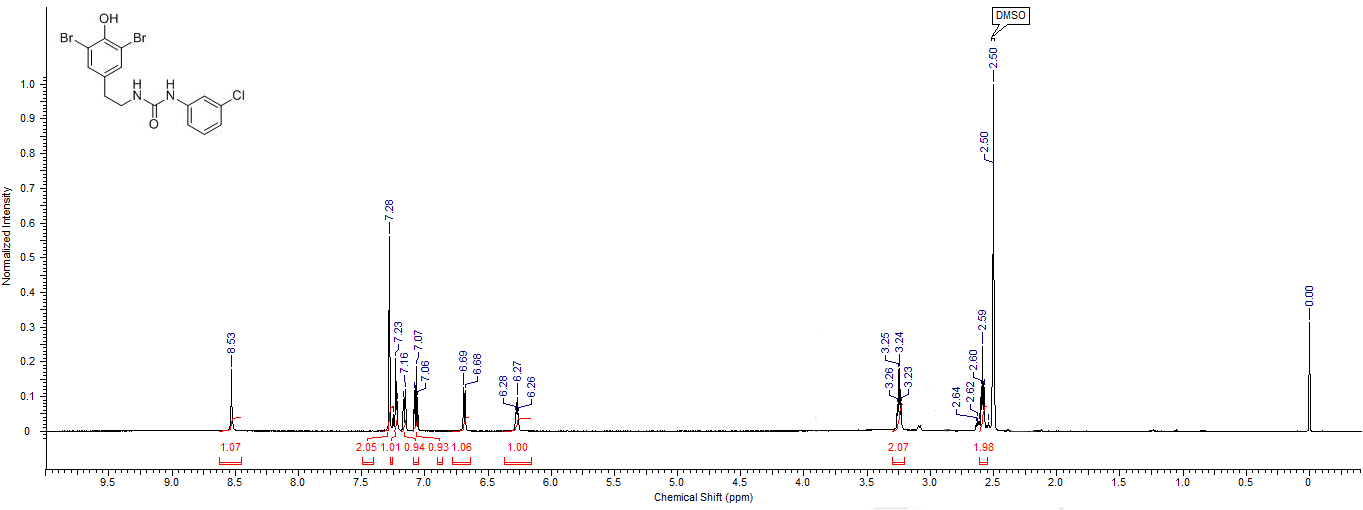
**

**4i ^1^H-NMR**

**
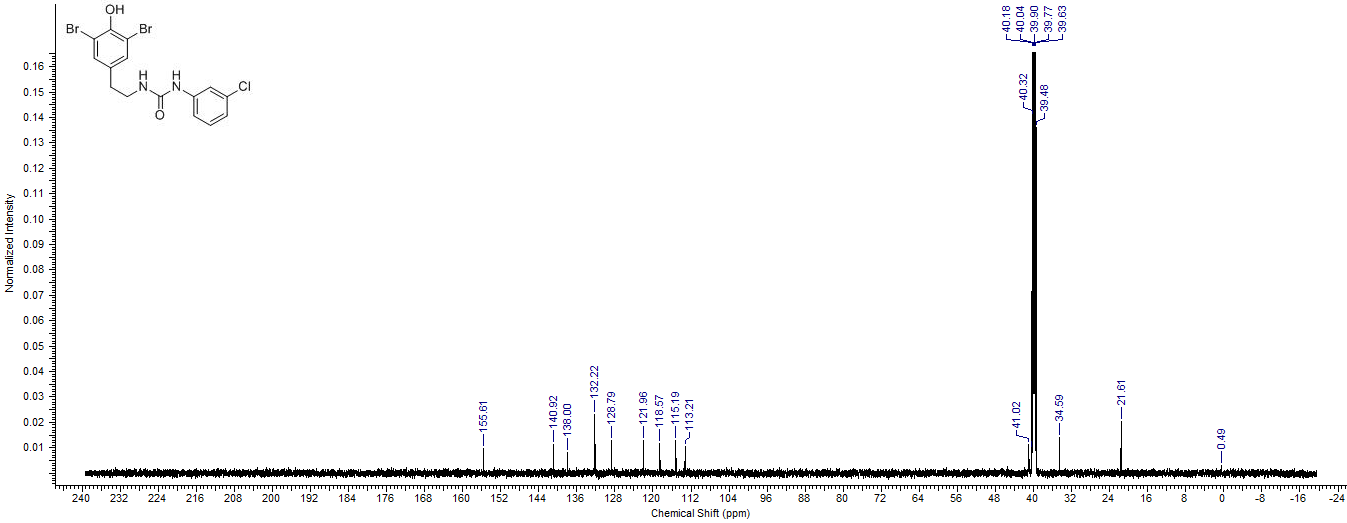
**

**4i ^13^C-NMR**

**
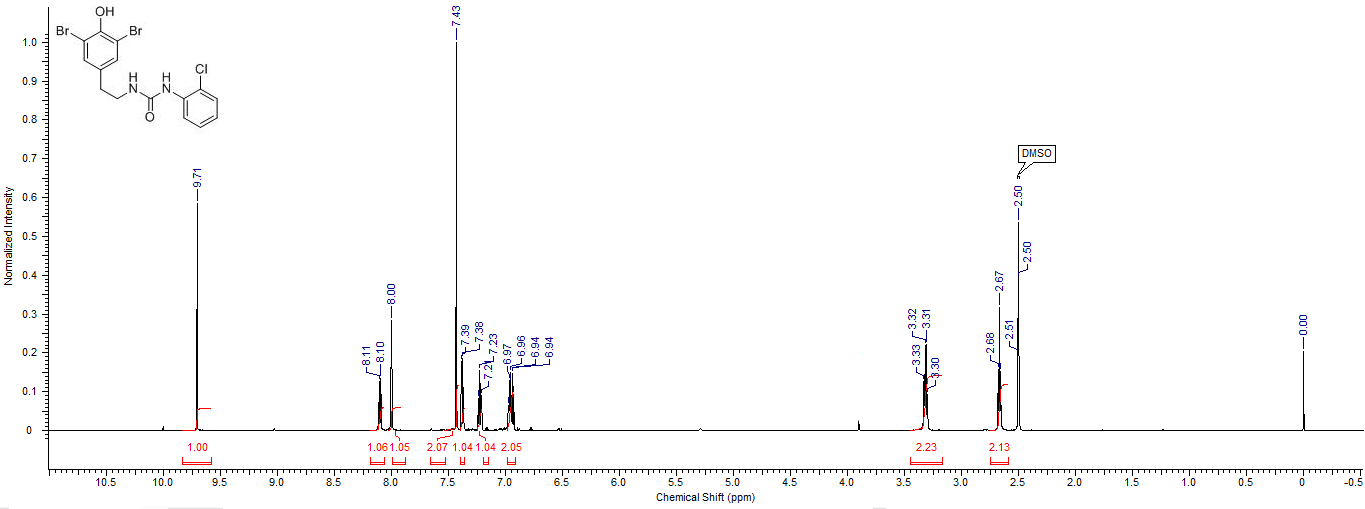
**

**4j ^1^H-NMR**

**
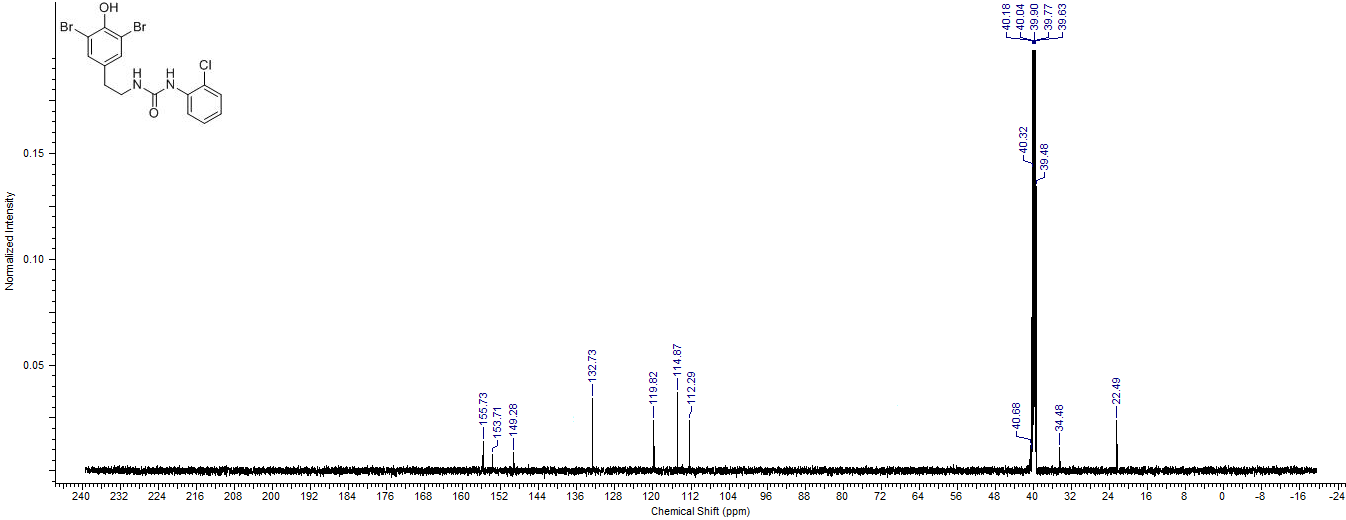
**

**4j ^13^C-NMR**

**
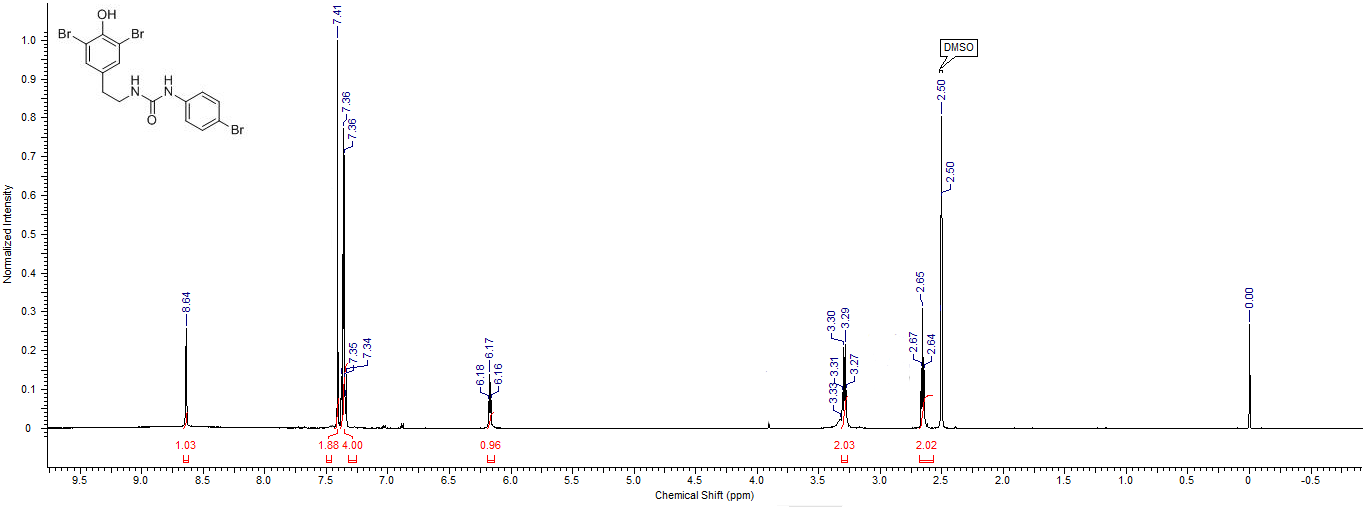
**

**4k ^1^H-NMR**

**
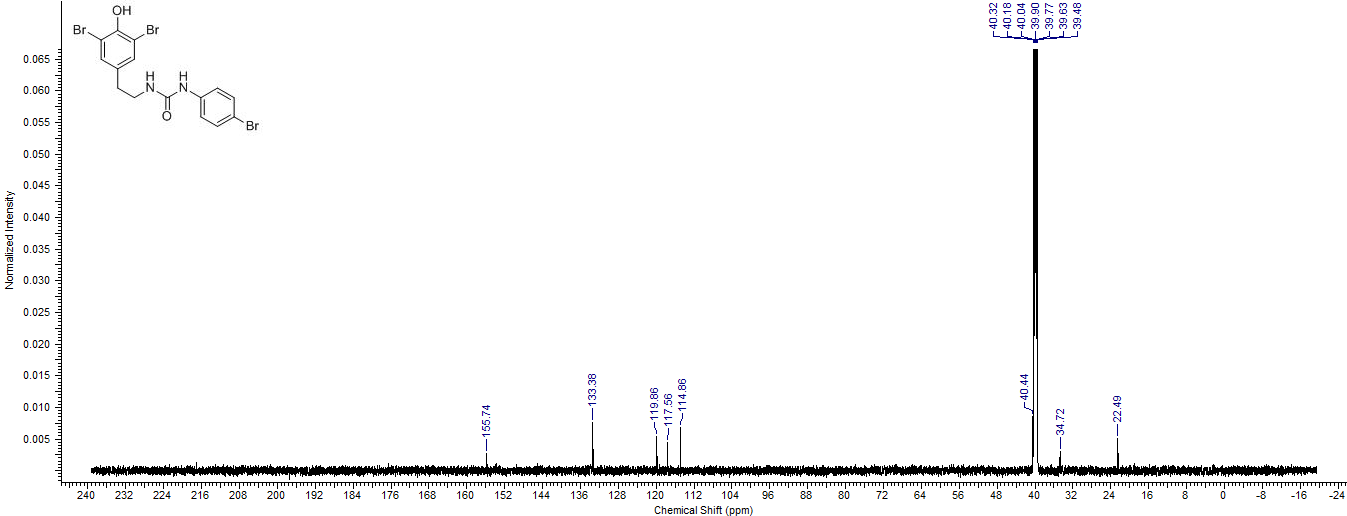
**

**4k ^13^C-NMR**

**
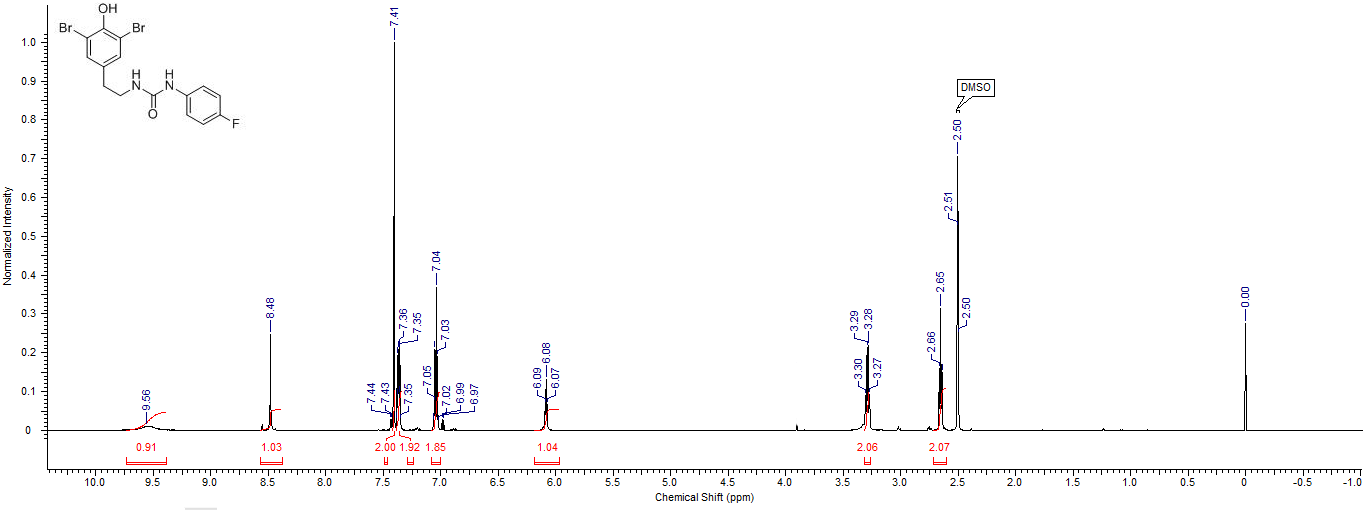
**

**4l ^1^H-NMR**

**
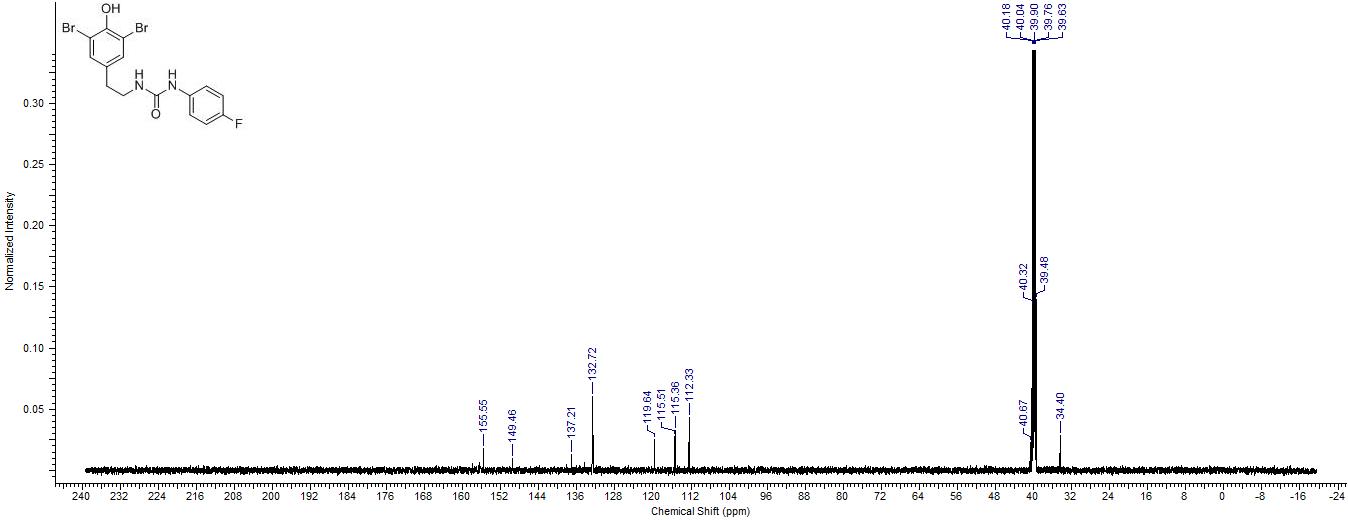
**

**4l ^13^C-NMR**

**
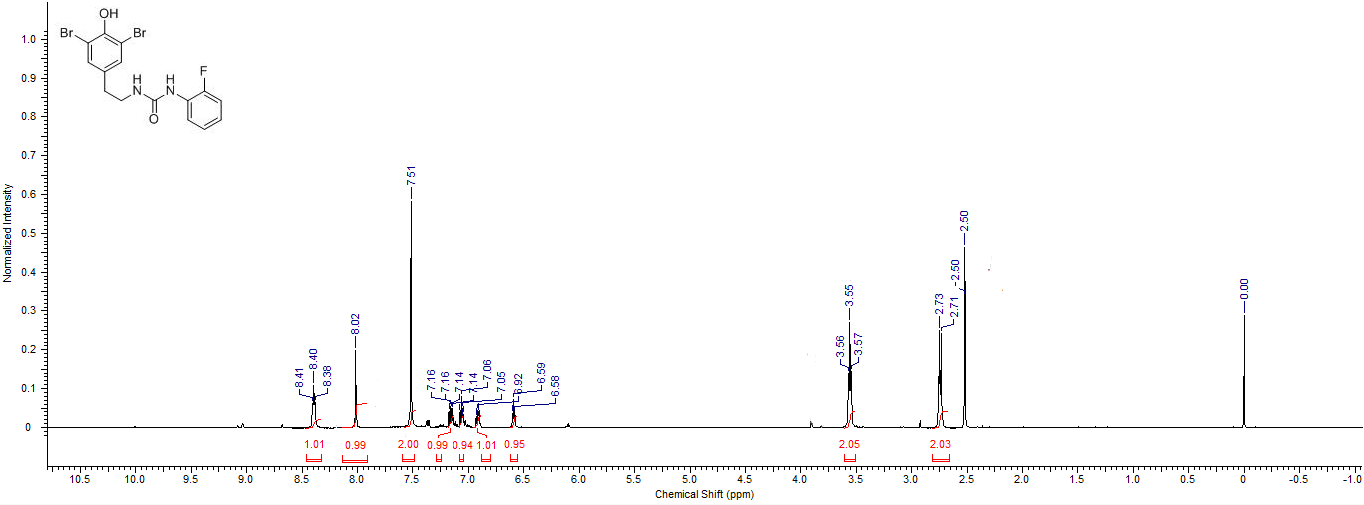
**

**4m ^1^H-NMR**

**
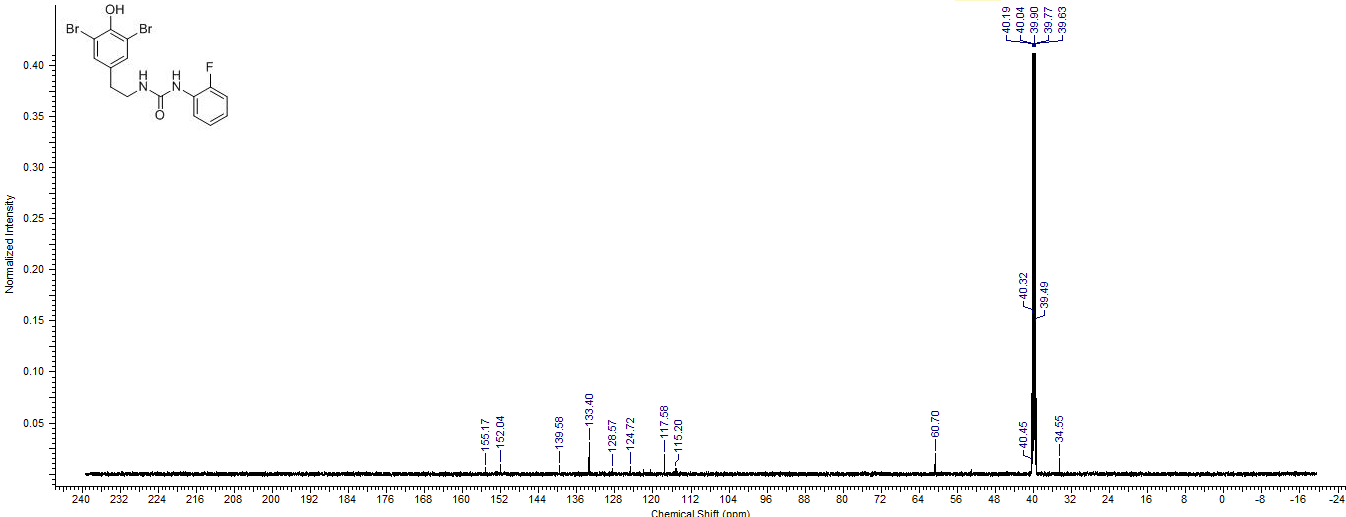
**

**4m ^13^C-NMR**

**
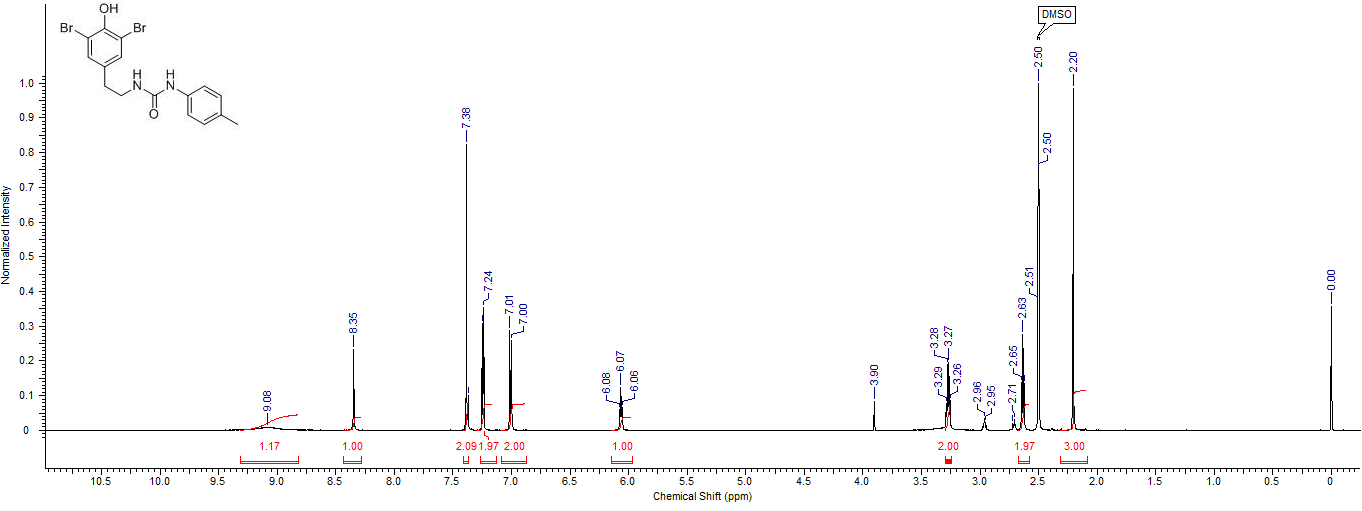
**

**4n ^1^H-NMR**

**
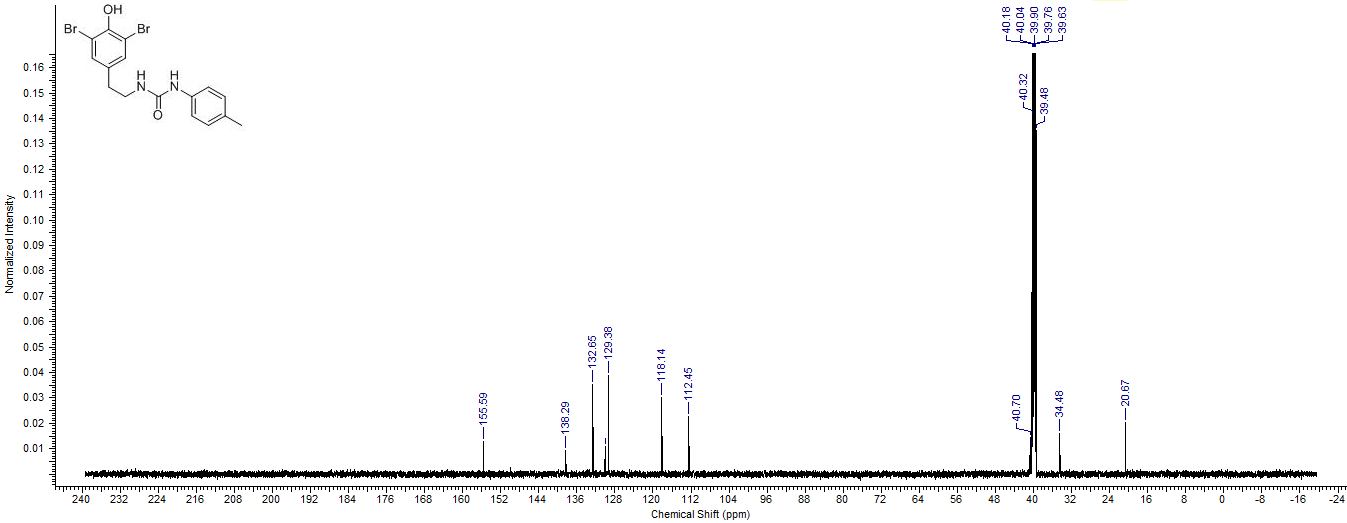
**

**4n ^13^C-NMR**

**
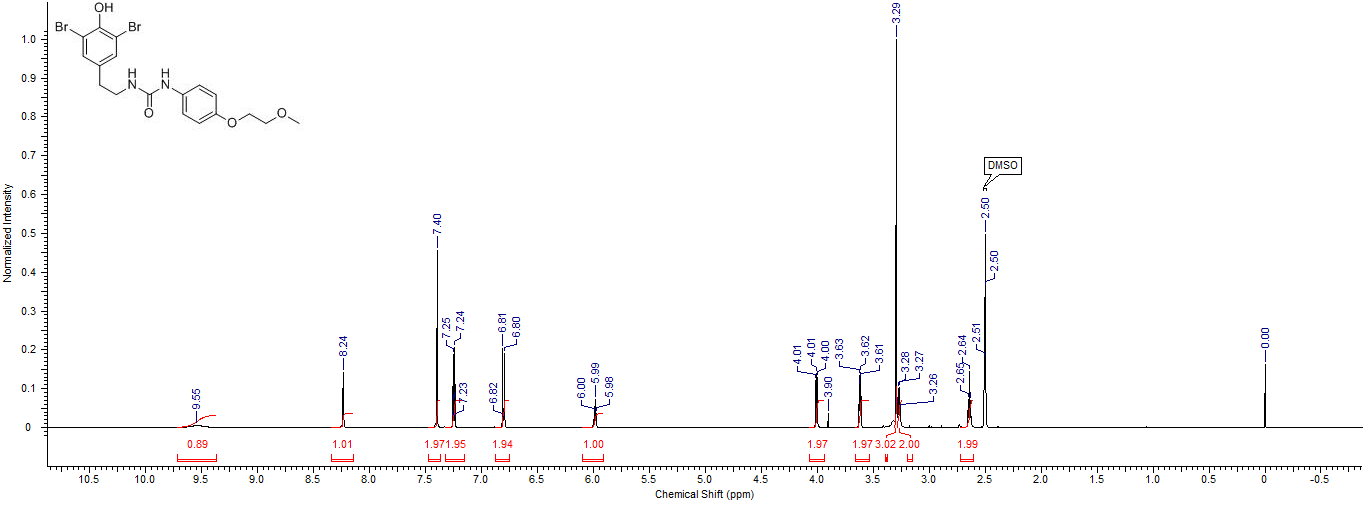
**

**4o ^1^H-NMR**

**
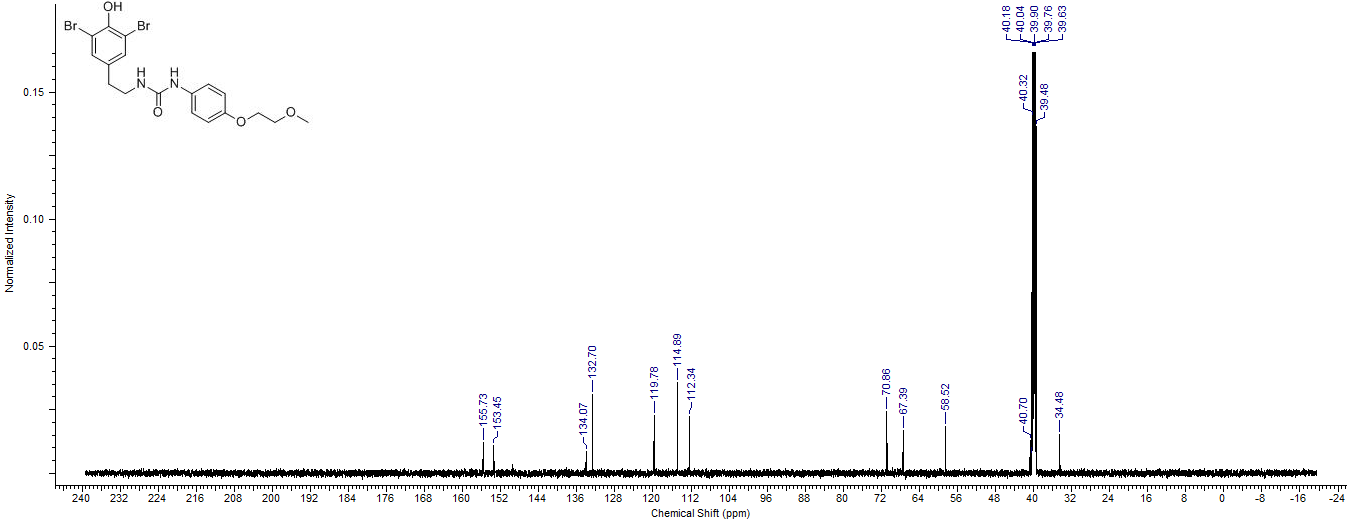
**

**4o ^13^C-NMR**
